# A Dynamic Connectome Supports the Emergence of Stable Computational Function of Neural Circuits through Reward-Based Learning

**DOI:** 10.1523/ENEURO.0301-17.2018

**Published:** 2018-04-24

**Authors:** David Kappel, Robert Legenstein, Stefan Habenschuss, Michael Hsieh, Wolfgang Maass

**Affiliations:** 1 Institute for Theoretical Computer Science, Graz University of Technology, 8010 Graz, Austria; 2 Bernstein Center for Computational Neuroscience, III Physikalisches Institut–Biophysik, Georg-August Universität, 37077 Göttingen, Germany; 3 Chair for Highly-Parallel VLSI-Systems and Neuromorphic Circuits, Technische Universität Dresden, 01069 Dresden, Germany

**Keywords:** reward-modulated STDP, spine dynamics, stochastic synaptic plasticity, synapse-autonomous processes, synaptic rewiring, task-irrelevant dimensions in motor control

## Abstract

Synaptic connections between neurons in the brain are dynamic because of continuously ongoing spine dynamics, axonal sprouting, and other processes. In fact, it was recently shown that the spontaneous synapse-autonomous component of spine dynamics is at least as large as the component that depends on the history of pre- and postsynaptic neural activity. These data are inconsistent with common models for network plasticity and raise the following questions: how can neural circuits maintain a stable computational function in spite of these continuously ongoing processes, and what could be functional uses of these ongoing processes? Here, we present a rigorous theoretical framework for these seemingly stochastic spine dynamics and rewiring processes in the context of reward-based learning tasks. We show that spontaneous synapse-autonomous processes, in combination with reward signals such as dopamine, can explain the capability of networks of neurons in the brain to configure themselves for specific computational tasks, and to compensate automatically for later changes in the network or task. Furthermore, we show theoretically and through computer simulations that stable computational performance is compatible with continuously ongoing synapse-autonomous changes. After reaching good computational performance it causes primarily a slow drift of network architecture and dynamics in task-irrelevant dimensions, as observed for neural activity in motor cortex and other areas. On the more abstract level of reinforcement learning the resulting model gives rise to an understanding of reward-driven network plasticity as continuous sampling of network configurations.

## Visual Abstract

**Figure:**
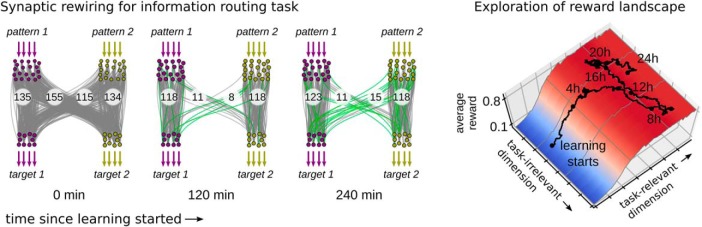

## Significance Statement

Networks of neurons in the brain do not have a fixed connectivity. We address the question how stable computational performance can be achieved by continuously changing neural circuits, and how these networks could even benefit from these changes. We show that the stationary distribution of network configurations provides a level of analysis where these issues can be addressed in a perspicuous manner. In particular, this theoretical framework allows us to address analytically the questions which rules for reward-gated synaptic rewiring and plasticity would work best in this context, and what impact different levels of activity-independent synaptic processes are likely to have. We demonstrate the viability of this approach through computer simulations and links to experimental data.

## Introduction

The connectome is dynamic: Networks of neurons in the brain rewire themselves on a time scale of hours to days (Holtmaat et al., 2005; Stettler et al., 2006; Holtmaat and Svoboda, 2009; Minerbi et al., 2009; Yang et al., 2009; Ziv and Ahissar, 2009; Kasai et al., 2010; Loewenstein et al., 2011, 2015; Rumpel and Triesch, 2016; Chambers and Rumpel, 2017; van Ooyen and Butz-Ostendorf, 2017). This synaptic rewiring manifests in the emergence and vanishing of dendritic spines (Holtmaat and Svoboda, 2009). Additional structural changes of established synapses are observable as a growth and shrinking of spine heads which take place even in the absence of neural activity (Yasumatsu et al., 2008). The recent study of Dvorkin and Ziv (2016), which includes in their Figure 8 a reanalysis of mouse brain data from Kasthuri et al. (2015), showed that this spontaneous component is surprisingly large, at least as large as the impact of pre- and postsynaptic neural activity. In addition, Nagaoka and colleagues provide direct evidence *in vivo* that the baseline turnover of dendritic spines is mediated by activity-independent intrinsic dynamics (Nagaoka et al., 2016). Furthermore, experimental data also suggest that task-dependent self-configuration of neural circuits is mediated by reward signals in Yagishita et al. (2014).

Other experimental data show that not only the connectome, but also the dynamics and function of neural circuits is subject to continuously ongoing changes. Continuously ongoing drifts of neural codes were reported in Ziv et al. (2013); Driscoll et al. (2017). Further data show that the mapping of inputs to outputs by neural networks that plan and control motor behavior are subject to a random walk on a slow time scale of minutes to days, that is conjectured to be related to stochastic synaptic rewiring and plasticity (Rokni et al., 2007; van Beers et al., 2013; Chaisanguanthum et al., 2014).

We address two questions that are raised by these data. (1) How can stable network performance be achieved in spite of the experimentally found continuously ongoing rewiring and activity-independent synaptic plasticity in neural circuits? (2) What could be a functional role of these processes?

**Figure 1.**
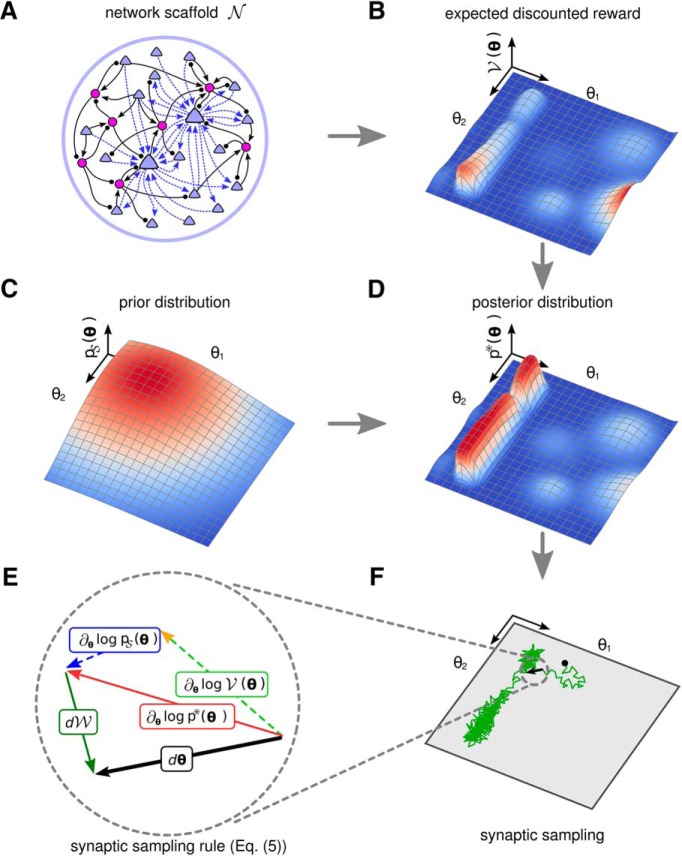
Illustration of the theoretical framework. ***A***, A neural network scaffold N of excitatory (blue triangles) and inhibitory (purple circles) neurons. Potential synaptic connections (dashed blue arrows) of only two excitatory neurons are shown to keep the figure uncluttered. Synaptic connections (black connections) from and to inhibitory neurons are assumed to be fixed for simplicity. ***B***, A reward landscape for two parameters $\text{θ}=\{\theta_{1},\theta_{2}\}$ with several local optima. Z-amplitude and color indicate the expected reward V(θ) for given parameters θ (*X-Y* plane). ***C***, Example prior that prefers small values for *θ*_1_ and *θ*_2_. ***D***, The posterior distribution $p^{*}(\text{θ})$ that results as product of the prior from ***C*** and the expected discounted reward of ***B***. ***E***, Illustration of the dynamic forces (plasticity rule Eq. 5) that act on θ in each sampling step dθ (black) while sampling from the posterior distribution. The deterministic term (red), which consists of the first two terms (prior and reward expectation) in Equation 5, is directed to the next local maximum of the posterior. The stochastic term dW (green) of Equation 5 has a random direction. ***F***, A single trajectory of policy sampling from the posterior distribution of ***D*** under Equation 5, starting at the black dot. The parameter vector θ fluctuates between different solutions and moves primarily along the task-irrelevant dimension *θ*_2_.

Similar as previously shown (Rokni et al., 2007; Statman et al., 2014; Loewenstein et al., 2015), we model spontaneous synapse-autonomous spine dynamics of each potential synaptic connection *i* through a stochastic process that modulates a corresponding parameter *θ_i_*. We provide in this article a rigorous mathematical framework for such stochastic spine dynamics and rewiring processes. Our analysis assumes that one can describe the network configuration, i.e., the current state of the dynamic connectome and the strengths of all currently functional synapses, at any time point by a vector θ that encodes the current values *θ_i_* for all potential synaptic connections *i*. The stochastic dynamics of this high-dimensional vector θ defines a Markov chain, whose stationary distribution (Fig. 1*D*) provides insight into questions that address the relation between properties of local synaptic processes and the computational function of a neural network.

Based on the well-studied paradigm for reward-based learning in neural networks, we propose the following answer to the first question: as long as most of the mass of this stationary distribution lies in regions or low-dimensional manifolds of the parameter space that produce good performance, stable network performance can be assured despite continuously ongoing movement of θ (Loewenstein et al., 2015). Our experimental results suggest that when a computational task has been learnt, most of the subsequent dynamics of θ takes place in task-irrelevant dimensions.

The same model also provides an answer to the second question: synapse-autonomous stochastic dynamics of the parameter vector θ enables the network not only to find in a high-dimensional region with good network performance but also to rewire the network to compensate for changes in the task. We analyze how the strength of the stochastic component of synaptic dynamics affects this compensation capability. We arrive at the conclusion that compensation works best for the task considered here if the stochastic component is as large as in experimental data (Dvorkin and Ziv, 2016).

On the more abstract level of reinforcement learning, our theoretical framework for reward-driven network plasticity suggests a new algorithmic paradigm for network learning: policy sampling. Compared with the familiar policy gradient learning (Williams, 1992; Baxter and Bartlett, 2000; Peters and Schaal, 2006), this paradigm is more consistent with experimental data that suggest a continuously ongoing drift of network parameters.

The resulting model for reward-gated network plasticity builds on the approach from Kappel et al. (2015) for unsupervised learning, that was only applicable to a specific neuron model and a specific Spike-timing-dependent plasticity rule. Since the new approach can be applied to arbitrary neuron models, in particular also to large data-based models of neural circuits and systems, it can be used to explore how data-based models for neural circuits and brain areas can attain and maintain a computational function.

## Results

We first address the design of a suitable theoretical framework for investigating the self-organization of neural circuits for specific computational tasks in the presence of spontaneous synapse-autonomous processes and rewards. There exist well-established models for reward-modulated synaptic plasticity, (Frémaux et al., 2010), where reward signals gate common rules for synaptic plasticity, such as STDP. But these rules are lacking two components that we need here: (1) an integration of rewiring with plasticity rules that govern the modulation of the strengths of already existing synaptic connections; and (2) a term that reflects the spontaneous synapse-autonomous component of synaptic plasticity and rewiring.

To illustrate our approach, we consider a neural network scaffold (Fig. 1*A*) with a large number of potential synaptic connections between excitatory neurons. Only a subset of these potential connections is assumed to be functional at any point in time.

If one allows rewiring then the concept of a neural network becomes problematic, since the definition of a neural network typically includes its synaptic connections. Hence, we refer to the set of neurons of a network, its set of potential synaptic connections, and its set of definite synaptic connections, such as in our case connections from and to inhibitory neurons (Fig. 1*A*), as a network scaffold. A network scaffold N together with a parameter vector θ that specifies a particular selection of functional synaptic connections out of the set of potential connections and particular synaptic weights for these defines a concrete neural network, to which we also refer as network configuration.

For simplicity we assume that only excitatory connections are plastic, but the model can be easily extended to also reflect plasticity of inhibitory synapses. For each potential synaptic connection *i*, we introduce a parameter *θ_i_* that describes its state both for the case when this potential connection *i* is currently not functional (this is the case when *θ_i_* ≤ 0) and when it is functional (i.e., *θ_i_* > 0). More precisely, *θ_i_* encodes the current strength or weight *w_i_* of this synaptic connection through the formula

(1) $$w_{i}\;=\;\{\begin{array}{ll} \exp(\theta_{i}-\theta_{0})\; & \text{if }\theta_{i}>0\;(functional\;synaptic\;connection)\\ 0\; & \text{if }\theta_{i}\leq 0\;(non-functional\;potential\;connection) \end{array},$$

with a positive offset parameter *θ*_0_ that regulates the initial strength of new functional synaptic connections (we set *θ*_0_ = 3 in our simulations).

**Figure 2.**
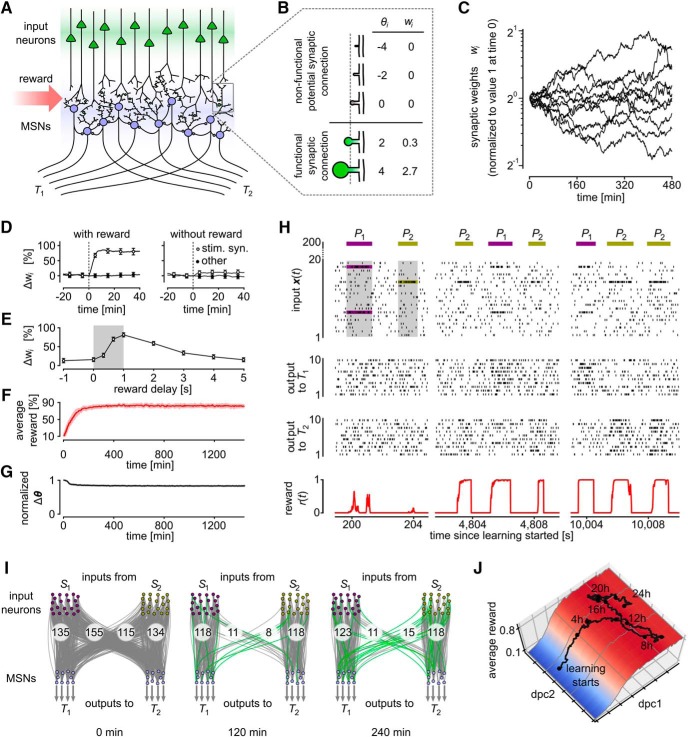
Reward-based routing of input patterns. ***A***, Illustration of the network scaffold. A population of 20 model MSNs (blue) receives input from 200 excitatory input neurons (green) that model cortical neurons. Potential synaptic connections between these two populations of neurons were subject to reward-based synaptic sampling. In addition, fixed lateral connections provided recurrent inhibitory input to the MSNs. The MSNs were divided into two groups, each projecting exclusively to one of two target areas *T*_1_ and *T*_2_. Reward was delivered whenever the network managed to route an input pattern *P_i_* primarily to that group of MSNs that projected to target area *T_i_*. ***B***, Illustration of the model for spine dynamics. Five potential synaptic connections at different states are shown. Synaptic spines are represented by circular volumes with diameters proportional to $\sqrt[{3}]{w_{i}}$ for functional connections, assuming a linear correlation between spine-head volume and synaptic efficacy *w_i_* (Matsuzaki et al., 2001). ***C***, Dynamics of weights *w_i_* in log scale for 10 potential synaptic connections *i* when the activity-dependent term $\frac{\partial }{\partial \theta_{i}}\log V(\text{θ})dt$ in Equation 5 is set equal to zero). As in experimental data (Holtmaat et al., 2006, their Fig. 2*I*) the dynamics is in this case consistent with an Ornstein–Uhlenbeck process in the logarithmic scale. Weight values are plotted relative to the initial value at time 0. ***D***, ***E***, Dynamics of a model synapse when a reward-modulated STDP pairing protocol as in Yagishita et al. (2014) was applied. ***D***, Reward delivery after repeated firing of the presynaptic neuron before the postsynaptic neuron resulted in a strong weight increase (left). This effect was reduced without reward (right) and prevented completely if no presynaptic stimulus was applied. Values in ***D***, ***E*** represent percentage of weight changes relative to the pairing onset time (dashed line, means ± SEM over 50 synapses). Compare with Yagishita et al. (2014), their Figure 1*F*,*G*. ***E***, Dependence of resulting changes in synaptic weights in our model as a function of the delay of reward delivery. Gray shaded rectangle indicates the time window of STDP pairing application. Reward delays denote time between paring and reward onset. Compare to Yagishita et al. (2014), their Figure 1*O*. ***F***, The average reward achieved by the network increased quickly during learning according to Equation 5 (mean over five independent trial runs; shaded area indicates SEM). ***G***, Synaptic parameters kept changing throughout the experiment in ***F***. The magnitude of the change of the synaptic parameter vector θ is shown (mean ± SEM as in ***F***; Euclidean norm, normalized to the maximum value). The parameter change peaks at the onset of learning but remains high (larger than 80% of the maximum value) even when stable performance has been reached. ***H***, Spiking activity of the network during learning. Activities of 20 randomly selected input neurons and all MSNs are shown. Three salient input neurons (belonging to pools *S*_1_ or *S*_2_ in ***I***) are highlighted. Most neurons have learnt to fire at a higher rate for the input pattern *P_j_* that corresponds to the target area *T_j_* to which they are projecting. Bottom, Reward delivered to the network. ***I***, Dynamics of network rewiring throughout learning. Snapshots of network configurations for the times *t* indicated below the plots are shown. Gray lines indicate active connections between neurons; connections that were not present at the preceding snapshot are highlighted in green. All output neurons and two subsets of input neurons that fire strongly in pattern *P*_1_ or *P*_2_ are shown (pools *S*_1_ and *S*_2_, 20 neurons each). Numbers denote total counts of functional connections between pools. The connectivity was initially dense and then rapidly restructured and became sparser. Rewiring took place all the time throughout learning. ***J***, Analysis of random exploration in task-irrelevant dimensions of the parameter space. Projection of the parameter vector θ to the two dPCA components that best explain the variance of the average reward. dpc1 explains >99.9% of the reward variance (dpc2 and higher dimensions <0.1%). A single trajectory of the high-dimensional synaptic parameter vector over 24 h of learning projected onto dpc1 and dpc2 is shown. Amplitude on the *y*-axis denotes the estimated average reward (in fractions of the total maximum achievable reward). After converging to a region of high reward (movement mainly along dpc1), network continues to explore task-irrelevant dimensions (movement mainly along dpc2).

The exponential function in Equation 1 turns out to be useful for relating the dynamics of *θ_i_* to experimental data on the dynamics of synaptic weights. The volume, or image brightness in Ca-imaging, of a dendritic spine is commonly assumed to be proportional to the strength *w_i_* of a synapse (Holtmaat et al., 2005). The logarithm of this estimate for *w_i_* was shown in Holtmaat et al. (2006), their Figure 2*I*, and also in Yasumatsu et al. (2008) and Loewenstein et al. (2011), to exhibit a dynamics similar to that of an Ornstein–Uhlenbeck process, i.e., a random walk in conjunction with a force that draws the random walk back to its initial state. Hence if *θ_i_* is chosen to be proportional to the logarithm of *w_i_*, it is justified to model the spontaneous dynamics of *θ_i_* as an Ornstein–Uhlenbeck process. This is done in our model, as we will explain after Equation 5 and demonstrate in Figure 2*C*. The logarithmic transformation also ensures that additive increments of *θ_i_* yield multiplicative updates of *w_i_*, which have been observed experimentally (Loewenstein et al., 2011).

Together, our model needs to create a dynamics for *θ_i_* that is not only consistent with experimental data on spontaneous spine dynamics, but is for the case *θ_i_* > 0 also consistent with rules for reward-modulated synaptic plasticity as in Frémaux et al. (2010). This suggests to look for plasticity rules of the form

(2) $$d\theta_{i}=\beta \times \left(\text{deterministic plasticity rule}\right)\times dt+\sqrt{2\beta T}dW_{i},$$

where the deterministic plasticity rule could for example be a standard reward-based plasticity rule. We will argue below that it makes sense to include also an activity-independent prior in this deterministic component of rule (2), both for functional reasons and to fit data on spontaneous spine dynamics. We will further see that when the activity-independent prior dominates, we obtain the Ornstein–Uhlenbeck process mentioned above. The stochastic term $dW_{i}$ in Equation 2 is an infinitesimal step of a random walk, more precisely for a Wiener process $W_{i}$. A Wiener process is a standard model for Brownian motion in one dimension (Gardiner, 2004). The term $\sqrt{2\beta T}$ scales the strength of this stochastic component in terms of a “temperature” *T* and a learning rate *β* and is chosen to be of a form that supports analogies to statistical physics. The presence of this stochastic term makes it unrealistic to expect that *θ_i_* converges to a particular value under the dynamics defined by Equation 2. In fact, in contrast to many standard differential equations, the stochastic differential equation or SDE (Eq. 2) does not have a single trajectory of *θ_i_* as solution but an infinite family of trajectories that result from different random walks.

We propose to focus, instead of the common analysis of the convergence of weights to specific values as invariants, on the most prominent invariant that a stochastic process can offer: the long-term stationary distribution of synaptic connections and weights. The stationary distribution of the vector θ of all synaptic parameters *θ_i_* informs us about the statistics of the infinitely many different solutions of a stochastic differential equation of the form of Equation 2. In particular, it informs us about the fraction of time at which particular values of θ will be visited by these solutions (for details, see Materials and Methods). We show that a large class of reward-based plasticity rules produce in the context of an equation of the form of Equation 2 a stationary distribution of θ that can be clearly related to reward expectation for the neural network, and hence to its computational function.

We want to address the question whether reward-based plasticity rules achieve in the context with other terms in Equation 2 that the resulting stationary distribution of network configurations has most of its mass on highly rewarded network configurations. A key observation is that if the first term on the right-hand-side of Equation 2 can be written for all potential synaptic connections *i* in the form $\frac{\partial }{\partial \theta_{i}}\log p^{*}(\text{θ})$, where $p^{*}(\text{θ})$ is some arbitrary given distribution and $\frac{\partial }{\partial \theta_{i}}$ denotes the partial derivative with respect to parameter *θ_i_*, then these stochastic processes

(3) $$d\theta_{i}=\beta \frac{\partial }{\partial \theta_{i}}\log p^{*}\left(\theta \right)dt+\sqrt{2\beta T}dW_{i}$$

give rise to a stationary distribution that is proportional to $p^{*}{\left(\text{θ}\right)}^{1/T}$. Hence, a rule for reward-based synaptic plasticity that can be written in the form $\frac{\partial }{\partial \theta_{i}}\log p^{*}(\text{θ})$, where $p^{*}(\text{θ})$ has most of its mass on highly rewarded network configurations θ, achieves that the network will spend most of its time in highly rewarded network configurations. This will hold even if the network does not converge to or stay in any particular network configuration θ (Fig. 1*D*,*F*). Furthermore, the role of the temperature *T* in Equation 3 becomes clearly visible in this result: if *T* is large the resulting stationary distribution flattens the distribution $p^{*}(\text{θ})$, whereas for 0 < *T* < 1 the network will remain for larger fractions of the time in those regions of the parameter space where $p^{*}(\text{θ})$ achieves its largest values. In fact, if the temperature *T* converges to 0, the resulting stationary distribution degenerates to one that has all of its mass on the network configuration θ for which $p^{*}(\text{θ})$ reaches its global maximum, as in simulated annealing (Kirkpatrick et al., 1983).

We will focus on target distributions $p^{*}(\text{θ})$ of the form

(4) $$p^{*}\left(\theta \right)\propto p_{S}\left(\theta \right)\times V\left(\theta \right),$$

where ∝ denotes proportionality up to a positive normalizing constant. $p_{S}(\text{θ})$ can encode structural priors of the network scaffold N. For example, it can encode a preference for sparsely connected networks. This happens when $p_{S}(\text{θ})$ has most of its mass near **0** (Fig. 1*C*). But it could also convey genetically encoded or previously learnt information, such as a preference for having strong synaptic connections between two specific populations of neurons. The term V(θ) in Equation 4 denotes the expected discounted reward associated with a given parameter vector θ (Fig. 1*B*). Equation 3 for the stochastic dynamics of parameters takes then the form

(5) $$d\theta_{i}=\beta \left(\frac{\partial }{\partial \theta_{i}}\log p_{S}\left(\theta \right)+\frac{\partial }{\partial \theta_{i}}\log V\left(\theta \right)\right)dt+\sqrt{2\beta T}dW_{i}.$$

When the term $\frac{\partial }{\partial \theta_{i}}\log V(\text{θ})$ vanishes, this equation models spontaneous spine dynamics. We will make sure that this term vanishes for all potential synaptic connections *i* that are currently not functional, i.e., where *θ_i_* ≤ 0. If one chooses a Gaussian distribution as prior $p_{S}\left(\text{θ}\right)$, the dynamics of Equation 5 amounts in the case $\frac{\partial }{\partial \theta_{i}}\log V\left(\theta \right)=0$ to an Ornstein–Uhlenbeck process. There is currently no generally accepted description of spine dynamics. Ornstein–Uhlenbeck dynamics has previously been proposed as a simple model for experimentally observed spontaneous spine dynamics (Loewenstein et al., 2011, 2015). Another proposed model uses a combination of multiplicative and additive stochastic dynamics (Statman et al., 2014; Rubinski and Ziv, 2015). We used in our simulations a Gaussian distribution that prefers small but nonzero weights for the prior $p_{S}\left(\text{θ}\right)$. Hence, our model (Eq. 5) is consistent with previous Ornstein–Uhlenbeck models for spontaneous spine dynamics.

Thus, altogether, we arrive at a model for the interaction of stochastic spine dynamics with reward where the usually considered deterministic convergence to network configurations θ that represent local maxima of expected reward V(θ) (e.g., to the local maxima in Fig. 1*B*) is replaced by a stochastic model. If the stochastic dynamics of θ is defined by local stochastic processes of the form of Equation 5, as indicated in Figure 1*E*, the resulting stochastic model for network plasticity will spend most of its time in network configurations θ where the posterior $p^{*}(\text{θ})$, illustrated in Figure 1*D*, approximately reaches its maximal value. This provides on the statistical level a guarantee of task performance, despite ongoing stochastic dynamics of all the parameters *θ_i_*.

### Reward-based rewiring and synaptic plasticity as policy sampling

We assume that all synapses and neurons in the network scaffold N receive reward signals *r*(*t*) at certain times *t*, corresponding for example to dopamine signals in the brain (for a recent discussion of related experimental data, see Collins and Frank, 2016). The expected discounted reward V(θ) that occurs in the second term of Equation 5 is the expectation of the time integral over all future rewards *r*(*t*), while discounting more remote rewards exponentially (Eq. 6). Figure 1*B* shows a hypothetical V(θ) landscape over two parameters *θ*_1_,*θ*_2_. The posterior $p^{*}(\text{θ})$ shown in Figure 1*D* is then proportional to the product of V(θ) (Fig. 1*B*) and the prior (Fig. 1*C*).

The computational behavior of the network configuration, i.e., the mapping of network inputs to network outputs that is encoded by the parameter vector θ, is referred to as a policy in the context of reinforcement learning theory. The parameters θ (and therefore the policy) are gradually changed through Equation 5 such that the expected discounted reward V(θ) is increased: The parameter dynamics follows the gradient of logV(θ), i.e., $\frac{d\theta_{i}}{dt}=\beta \frac{\partial }{\partial \theta_{i}}\log V(\text{θ})$, where *β* > 0 is a small learning rate. When the parameter dynamics is given solely by the second term in the parenthesis of Equation 5, $\frac{\partial }{\partial \theta_{i}}\log V(\text{θ})$, we recover for the case *θ_i_* > 0 deterministic policy gradient learning (Williams, 1992; Baxter and Bartlett, 2000; Peters and Schaal, 2006).

For a network scaffold N of spiking neurons, the derivative $\frac{\partial }{\partial \theta_{i}}\log V(\text{θ})$ gives rise to synaptic updates at a synapse *i* that are essentially given by the product of the current reward signal *r*(*t*) and an eligibility trace that depends on pre- or postsynaptic firing times (see Materials and Methods, Synaptic dynamics for the reward-based synaptic sampling model). Such plasticity rules have previously been proposed (Seung, 2003; Xie and Seung, 2004; Pfister et al., 2006; Florian, 2007; Izhikevich, 2007; Legenstein et al., 2008; Urbanczik and Senn, 2009). For nonspiking neural networks, a similar update rule was first introduced by Williams and termed the REINFORCE rule (Williams, 1992).

In contrast to policy gradient, reinforcement learning in the presence of the stochastic last term in Equation 5 cannot converge to any network configuration. Instead, the dynamics of Equation 5 produces continuously changing network configurations, with a preference for configurations that both satisfy constraints from the prior $p_{S}(\text{θ})$ and provide a large expected reward V(θ) (Fig. 1*D*,*F*). Hence this type of reinforcement learning samples continuously from a posterior distribution of network configurations. This is rigorously proven in Theorem 1 of Methods. We refer to this reinforcement learning model as policy sampling, and to the family of reward-based plasticity rules that are defined by Equation 5 as reward-based synaptic sampling.

Another key difference to previous models for reward-gated synaptic plasticity and policy gradient learning is, apart from the stochastic last term of Equation 5, that the deterministic first term of Equation 5 also contains a reward-independent component $\frac{\partial }{\partial \theta_{i}}\log p_{S}(\text{θ})$ that arises from a prior $p_{S}(\text{θ})$ for network configurations. In our simulations we consider a simple Gaussian prior $p_{S}(\text{θ})$ with mean **0** that encodes a preference for sparse connectivity (Eq. 17).

**Figure 3.**
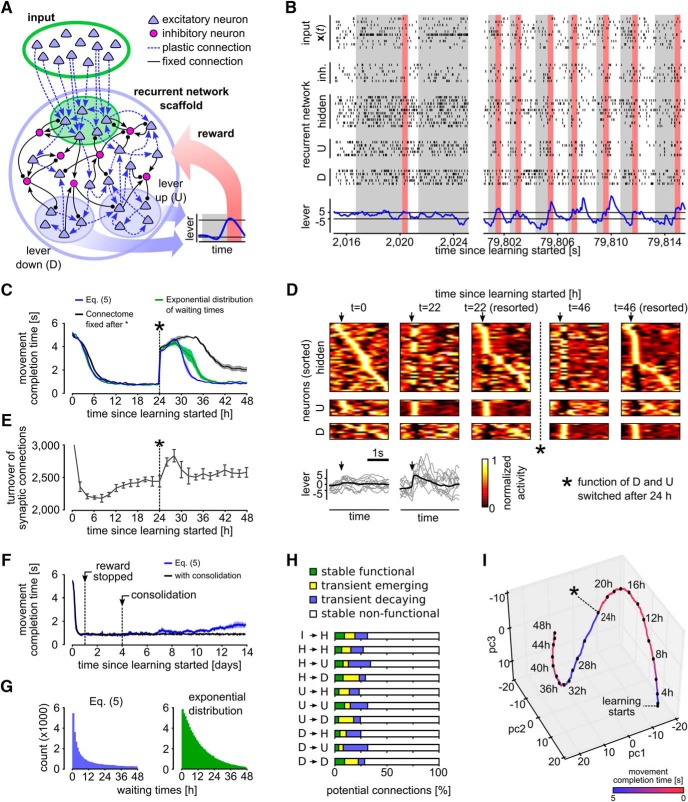
Reward-based self-configuration and compensation capability of a recurrent neural network. ***A***, Network scaffold and task schematic. Symbol convention as in Figure 1*A*. A recurrent network scaffold of excitatory and inhibitory neurons (large blue circle); a subset of excitatory neurons received input from afferent excitatory neurons (indicated by green shading). From the remaining excitatory neurons, two pools D and U were randomly selected to control lever movement (blue shaded areas). Bottom inset, Stereotypical movement that had to be generated to receive a reward. ***B***, Spiking activity of the network at learning onset and after 22 h of learning. Activities of random subsets of neurons from all populations are shown (hidden: excitatory neurons of the recurrent network, which are not in pool D or U). Bottom, Lever position inferred from the neural activity in pools D and U. Rewards are indicated by red bars. Gray shaded areas indicate cue presentation. ***C***, Task performance quantified by the average time from cue presentation onset to movement completion. The network was able to solve this task in <1 s on average after ∼8 h of learning. A task change was introduced at time 24 h (asterisk; function of D and U switched), which was quickly compensated by the network. Using a simplified version of the learning rule, where the reintroduction of nonfunctional potential connections was approximated using exponentially distributed waiting times (green), yielded similar results (see also ***E***). If the connectome was kept fixed after the task change at 24 h, performance was significantly worse (black). ***D***, Trial-averaged network activity (top) and lever movements (bottom). Activity traces are aligned to movement onsets (arrows); *y*-axis of trial-averaged activity plots are sorted by the time of highest firing rate within the movement at various times during learning: sorting of the first and second plot is based on the activity at *t* = 0 h, third and fourth by that at *t* = 22 h, fifth is resorted by the activity at *t* = 46 h. Network activity is clearly restructured through learning with particularly stereotypical assemblies for sharp upward movements. Bottom: average lever movement (black) and 10 individual movements (gray). ***E***, Turnover of synaptic connections for the experiment shown in ***D***; *y*-axis is clipped at 3,000. Turnover rate during the first 2 h was around 12,000 synapses (∼25%) and then decreased rapidly. Another increase in spine turnover rate can be observed after the task change at time 24 h. ***F***, Effect of forgetting due to parameter diffusion over 14 simulated days. Application of reward was stopped after 24 h when the network had learned to reliably solve the task. Parameters subsequently continue to evolve according to the SDE (Eq. 5). Onset of forgetting can be observed after day 6. A simple consolidation mechanism triggered after 4 days reliably prevents forgetting. ***G***, Histograms of time intervals between disappearance and reappearance of synapses (waiting times) for the exact (upper plot) and approximate (lower plot) learning rule. ***H***, Relative fraction of potential synaptic connections that were stably nonfunctional, transiently decaying, transiently emerging or stably function during the relearning phase for the experiment shown in ***D***. ***I***, PCA of a random subset of the parameters *θ_i_*. The plot suggests continuing dynamics in task-irrelevant dimensions after the learning goal has been reached (indicated by red color). When the function of the neuron pools U and D was switched after 24 h, the synaptic parameters migrated to a new region. All plots show means over five independent runs (error bars: SEM).

It is important that the dynamics of disconnected synapses, i.e., of synapses *i* with *θ_i_* ≤ 0 or equivalently *w_i_* = 0, does not depend on pre-/postsynaptic neural activity or reward since nonfunctional synapses do not have access to such information. This is automatically achieved through our ansatz $\frac{\partial }{\partial \theta_{i}}\log V(\text{θ})$ for the reward-dependent component in Equation 5, since a simple derivation shows that it entails that the factor *w_i_* appears in front of the term that depends on pre- and postsynaptic activity (Eq. 15). Instead, the dynamics of *θ_i_* depends for *θ_i_* ≤ 0 only on the prior and the stochastic term $dW_{i}$. This results in a distribution over waiting times between downwards and upwards crossing of the threshold *θ_i_* = 0 that was found to be similar to the distribution of inter-event times of a Poisson point process (for a detailed analysis, see Ding and Rangarajan, 2004). This theoretical result suggest a simple approximation of the dynamics of Equation 5 for currently nonfunctional synaptic connections, where the process of Equation 5 is suspended whenever *θ_i_* becomes negative, and continued with *θ_i_* = 0 after a waiting time that is drawn from an exponential distribution. As in Deger et al. (2016), this can be realized by letting a nonfunctional synapse become functional at any discrete time step with some fixed probability (Poisson process). We have compared in Figure 3*C* the resulting learning dynamics of the network for this simple approximation with that of the process defined by Equation 5.

### Task-dependent routing of information through the interaction of stochastic spine dynamics with rewards

Experimental evidence about gating of spine dynamics by reward signals in the form of dopamine is available for the synaptic connections from the cortex to the entrance stage of the basal ganglia, the medium spiny neurons (MSNs) in the striatum (Yagishita et al., 2014). They report that the volumes of their dendritic spines show significant changes only when pre- and postsynaptic activity is paired with precisely timed delivery of dopamine (Yagishita et al., 2014; Fig. 1*E–G*,*O*). More precisely, an STDP pairing protocol followed by dopamine uncaging induced strong LTP in synapses onto MSNs, whereas the same protocol without dopamine uncaging lead only to a minor increase of synaptic efficacies.

MSNs can be viewed as readouts from a large number of cortical areas, that become specialized for particular motor functions, e.g., movements of the hand or leg. We asked whether reward gating of spine dynamics according to the experimental data of Yagishita et al. (2014) can explain such task dependent specialization of MSNs. More concretely, we asked whether it can achieve that two different distributed activity patterns *P*_1_, *P*_2_ of upstream neurons in the cortex get routed to two different ensembles of MSNs, and thereby to two different downstream targets *T*_1_ and *T*_2_ of these MSNs (Fig. 2*A*,*H*,*I*). We assumed that for each upstream activity pattern *P_j_* a particular subset *S_j_* of upstream neurons is most active, *j* = 1, 2. Hence this routing task amounted to routing synaptic input from *S_j_* to those MSNs that project to downstream neuron *T_j_*.

We applied to all potential synaptic connections *i* from upstream neurons to MSNs a learning rule according to Equation 5, more precisely, the rule for reward-gated STDP (Eqs. 15, 16, 18) that results from this general framework. The parameters of the model were adapted to qualitatively reproduce the results from Yagishita et al. (2014), their Figure 1*F*,*G*, when the same STDP protocol was applied to our model (Fig. 2*D*,*E*). The parameter values are reported in Table 1. If not stated otherwise, we applied these parameters in all following experiments. In Role of the prior distribution (see Materials and Methods), we further analyze the impact of different prior distributions on task performance and network connectivity.

Our simple model consisted of 20 inhibitory model MSNs with lateral recurrent connections. These received excitatory input from 200 input neurons. The synapses from input neurons to model MSNs were subject to our plasticity rule. Multiple connections were allowed between each pair of input neuron and MSNs (see Materials and Methods). The MSNs were randomly divided into two assemblies, each projecting exclusively to one of two downstream target areas *T*_1_ and *T*_2_. Cortical input x(t) was modeled as Poisson spike trains from the 200 input neurons with instantaneous rates defined by two prototype rate patterns *P*_1_ and *P*_2_ (Fig. 2*H*). The task was to learn to activate *T*_1_-projecting neurons and to silence *T*_2_-projecting neurons whenever pattern *P*_1_ was presented as cortical input. For pattern *P*_2_, the activation should be reversed: activate *T*_2_-projecting neurons and silence those projecting to *T*_1_. This desired function was defined through a reward signal *r*(*t*) that was proportional to the ratio between the mean firing rate of MSNs projecting to the desired target and that of MSNs projecting to the nondesired target area (see Materials and Methods).

Figure 2*H* shows the firing activity and reward signal of the network during segments of one simulation run. After ∼80 min of simulated biological time, each group of MSNs had learned to increase its firing rate when the activity pattern *P_j_* associated with its projection target *T_j_* was presented. Figure 2*F* shows the average reward throughout learning. After 3 h of learning ∼82% of the maximum reward was acquired on average, and this level was maintained during prolonged learning.

Figure 2*G* shows that the parameter vector θ kept moving at almost the same speed even after a high plateau of rewards had been reached. Hence these ongoing parameter changes took place in dimensions that were irrelevant for the reward-level.

Figure 2*I* provides snapshots of the underlying “dynamic connectome” (Rumpel and Triesch, 2016) at different points of time. New synaptic connections that were not present at the preceding snapshot are colored green. One sees that the bulk of the connections maintained a solution of the task to route inputs from *S*_1_ to target area *T*_1_ and inputs from *S*_2_ to target area *T*_2_. But the identity of these connections, a task-irrelevant dimension, kept changing. In addition, the network always maintained some connections to the currently undesired target area, thereby providing the basis for a swift built-up of these connections if these connections would suddenly also become rewarded.

We further examine the exploration along task-irrelevant dimensions in Figure 2*J*. Here, the high-dimensional parameter vector over a training experiment of 24 h projected to the first two components of the demixed principal component analysis (dPCA) that best explain the variance of the average reward is shown (see Materials and Methods; Kobak et al., 2016). The first component (dpc1) explains >99.9% of the variance. Movement of the parameter vector mainly takes place along this dimensions during the first 4 h of learning. After the performance has converged to a high value, exploration continues along other components (dpc2, and higher components) that explain <0.1% of the average reward variance.

This simulation experiment showed that reward-gated spine dynamics as analyzed previously (Yagishita et al., 2014) is sufficiently powerful from the functional perspective to rewire networks so that each signal is delivered to its intended target.

### A model for task-dependent self-configuration of a recurrent network of excitatory and inhibitory spiking neurons

We next asked, whether our simple integrated model for reward-modulated rewiring and synaptic plasticity of neural circuits according to Equation 5 could also explain the emergence of specific computations in recurrent networks of spiking neurons. As paradigm for a specific computational task we took a simplified version of the task that mice learned to carry out in the experimental setup of Peters et al. (2014). There a reward was given whenever a lever was pressed within a given time window indicated by an auditory cue. This task is particular suitable for our context, since spine turnover and changes of network activity were continuously monitored in Peters et al. (2014), while the animals learned this task.

We adapted the learning task of Peters et al. (2014) in the following way for our model (Fig. 3*A*). The beginning of a trial was indicated through the presentation of a cue input pattern x(t): a fixed, randomly generated rate pattern for all 200 input neurons that lasted until the task was completed, but at most 10 s. As network scaffold N, we took a generic recurrent network of excitatory and inhibitory spiking neurons with connectivity parameters for connections between excitatory and inhibitory neurons according to data from layer 2/3 in mouse cortex (Avermann et al., 2012). The network consisted of 60 excitatory and 20 inhibitory neurons (Fig. 3*A*). Half of the excitatory neurons could potentially receive synaptic connections from the 200 excitatory input neurons. From the remaining 30 neurons, we randomly selected one pool D of 10 excitatory neurons to cause downwards movements of the lever, and another pool U of 10 neurons for upwards movements. We refer to the 40 excitatory neurons that were not members of D or U as hidden neurons. All excitatory synaptic connections from the external input (cue) and between the 60 excitatory neurons (including those in the pools D and U) in the network were subjected to reward-based synaptic sampling.

To decode the lever position, we filtered the population spikes of D and U with a smoothing kernel. The filtered population spikes of D were then subtracted from those of U to determine the lever position (see Methods for details). When the lever position crossed the threshold +5 after first crossing a lower threshold -5 (Fig. 3*A*,*B*, black horizontal lines) within 10 s after cue onset a 400-ms reward window was initiated during which *r*(*t*) was set to 1 (Fig. 3*B*, red vertical bars). Unsuccessful trials were aborted after 10 s and no reward was delivered. After each trial a brief holding phase of random length was inserted, during which input neurons were set to a background input rate of 2 Hz.

Thus, the network had to learn without any guidance, except for the reward in response to good performance, to create after the onset of the cue first higher firing in pool D, and then higher firing in pool U. This task was challenging, since the network had no information which neurons belonged to pools D and U. Moreover, the synapses did not “know” whether they connected to hidden neurons, neurons within a pool, hidden neurons and pool-neurons, or input neurons with other neurons. The plasticity of all these different synapses was gated by the same global reward signal. Since the pools D and U were not able to receive direct synaptic connections from the input neurons, the network also had to learn to communicate the presence of the cue pattern via disynaptic connections from the input neurons to these pools.

Network responses before and after learning are shown in Figure 3*B*. Initially, the rewarded goal was only reached occasionally, while the turnover of synaptic connections (number of synaptic connections that became functional or became nonfunctional in a time window of 2 h) remained very high (Fig. 3*E*). After ∼3 h, performance improved drastically (Fig. 3*C*), and simultaneously the turnover of synaptic connections slowed down (Fig. 3*E*). After learning for 8 h, the network was able to solve the task in most of the trials, and the average trial duration (movement completion time) had decreased to <1 s (851 ± 46 ms; Fig. 3*C*). Improved performance was accompanied by more stereotyped network activity and lever movement patterns as in the experimental data of Peters et al. (2014): compare our Figure 3*D* with Figures 1*B* and 2*J* of Peters et al. (2014). In Figure 3*D*, we show the trial-averaged activity of the 60 excitatory neurons before and after learning for 22 h. The neurons are sorted in the first two plots of Figure 3*D* by the time of maximum activity after movement onset times before learning, and in the 3rd plot resorted according to times of maximum activity after 22 h of learning (see Materials and Methods). These plots show that reward-based learning led to a restructuring of the network activity: an assembly of neurons emerged that controlled a sharp upwards movement. Also, less background activity was observed after 22 h of learning, in particular for neurons with early activity peaks. Fig. 3*D*, lower panels, shows the average lever movement and 10 individual movement traces at the beginning and after 22 h of learning. Similar as in Peters et al. (2014), the lever movements became more stereotyped during learning, featuring a sharp upwards movement at cue onset followed by a slower downwards movement in preparation for the next trial.

The synaptic parameter drifts due to stochastic differential Equation 5 inherently lead to forgetting. In Figure 3*F*, we tested this effect by running a very long experiment over 14 simulated days. After 24 h, when the network had learned to reliably solve the task, we stopped the application of the reward but continued the synaptic dynamics. We found that the task could be reliably recalled for >5 d. Onset of forgetting was observed after day 6. We wondered whether a simple consolidation mechanism could prevent forgetting in our model. To test this, we used the prior distribution $p_{S}\left(\text{θ}\right)$ to stabilize the synaptic parameters. After four simulated days we set the mean of the before the current value of the synaptic parameters and reduced the variance, while continuing the synaptic dynamics with the same temperature. A similar mechanism for synaptic consolidation has been recently suggested previously (Kirkpatrick et al., 2017). This mechanism reliably prevents forgetting in our model throughout the simulation time of 14 d. We conclude that the effect of forgetting is quite mild in our model and can be further suppressed by a consolidation mechanism that stabilizes synapses on longer timescales.

Next, we tested whether similar results could be achieved with a simplified version of the stochastic synapse dynamics while a potential synaptic connection *i* is nonfunctional, i.e., *θ_i_* ≤ 0. Equation 5 defines for such nonfunctional synapses an Ornstein–Uhlenbeck process, which yields a heavy-tailed distribution for the waiting time until reappearance (Fig. 3*G*, left). We tested whether similar learning performance can be achieved if one approximates the distribution by an exponential distribution, for which we chose a mean of 12 h. The small distance between the blue and green curve in Figure 3*C* shows that this is in fact the case for the overall computational task that includes a task switch at 24 h that we describe below. Compensation for the task switch was slightly slower when the approximating exponential distribution was used, but the task performance converged to the same result as for the exact rule. This holds despite the fact that the approximating exponential distribution is less heavy tailed (Fig. 3*G*, right). Together, these results show that rewiring and synaptic plasticity according to Equation 5 yields self-organization of a generic recurrent network of spiking neurons so that it can control an arbitrarily chosen motor control task.

### Compensation for network perturbations

We wondered whether this model for the task of Peters et al. (2014) would in addition be able to compensate for a drastic change in the task, an extra challenge that had not been considered in the experiments of Peters et al. (2014). To test this, we suddenly interchanged the actions that were triggered by the pools D and U at 24 h after learning had started. D now caused upwards and U downwards lever movement.

We found that our model compensated immediately (see the faster movement in the parameter space depicted in Fig. 3*H*) for this perturbation and reached after ∼8 h a similar performance level as before (Fig. 3*C*). The compensation was accompanied by a substantial increase in the turnover of synaptic connections (Fig. 3*E*). This observation is similar to findings from experiments that involve learning a new task (Xu et al., 2009). The turnover rate also remained slightly elevated during the subsequent learning period. Furthermore, a new assembly of neurons emerged that now triggered a sharp onset of activity in the pool D (compare the activity neural traces t = 22 h and t = 46 h; Fig. 3*D*). Another experimentally observed phenomenon that occurred in our model were drifts of neural codes, which happened also during phases of the experiment without perturbations. Despite these drifts, the task performance stayed constant, similar to experimental data in Driscoll et al. (2017 see Relative contributions of spontaneous and activity-dependent synaptic processes).

In Figure 3*H*, we further analyzed the profile of synaptic turnover for the different populations of the network scaffold in Figure 3*A*. The synaptic parameters were measured immediately before the task change at 24 h and compared to the connectivity after compensation at 48 h for the experiment shown in Figure 3*C*, blue. Most synapses (66–75%) were nonfunctional before and after the task change (stable nonfunctional). Approximately 20% of the synapses changed their behavior and either became functional or nonfunctional. Most prominently a large fraction (21.9%) of the synapses from hidden neurons to U became nonfunctional while only few (5.9%) new connections were introduced. The connections from hidden to D showed the opposite behavior. This modification of the network connectome reflects the requirement to reliably route information about the presence of the cue pattern encoded in the activity of hidden neurons to the pool D (and not to U) to initiate the lever movement after the task change.

If rewiring was disabled after the task change at 24 h the compensation was significantly delayed and overall performance declined (Fig. 3*C*, black curve). Here, we disallowed any turnover of potential synaptic connections such that the connectivity remained the same after 24 h. This result suggests that rewiring is necessary for adapting to the task change. We then asked whether rewiring is also necessary for the initial learning of the task. To answer this question, we performed a simulation where the network connectivity was fixed from the beginning. We found that initial task performance was not significantly worse compared to the setup with rewiring. This indicates that at least for this task, rewiring is necessary for compensating task switches, but not for initial task learning. We expect however that this is not the case for more complex tasks, as indicated by a recent study that used artificial neural networks (Bellec et al., 2017).

A structural difference between stochastic learning models such as policy sampling and learning models that focus on convergence of parameters to a (locally) optimal setting becomes apparent when one tracks the temporal evolution of the network parameters θ over larger periods of time during the previously discussed learning process (Fig. 3*I*). Although performance no longer improved after 5 h, both network connectivity and parameters kept changing in task-irrelevant dimensions. For Figure 3*I*, we randomly selected 5% of the roughly 47,000 parameters *θ_i_* and plotted the first three principal components of their dynamics. The task change after 24 h caused the parameter vector θ to migrate to a new region within ∼8 h of continuing learning (see Materials and Methods the projected parameter dynamics is further analyzed). Again, we observe that policy sampling keeps exploring different equally good solutions after the learning process has reached stable performance.

To further investigate the impact of the temperature parameter *T* on the magnitude of parameter changes, we measured the amplitudes of parameter changes for different values of *T*. We recorded the synaptic parameters every 20 min and measured the average Euclidean distance between successive snapshots of the parameter vectors. We found that a temperature of *T* = 0.1 increased the amplitude of parameter changes by around 150% compared to the case of *T* = 0. A temperature of *T* = 0.5 resulted in an increase of around 400%. Since this increase is induced by additional noise on parameter changes, it can be attributed to enhanced exploration in parameters space.

### Role of the prior distribution

**Figure 4.**
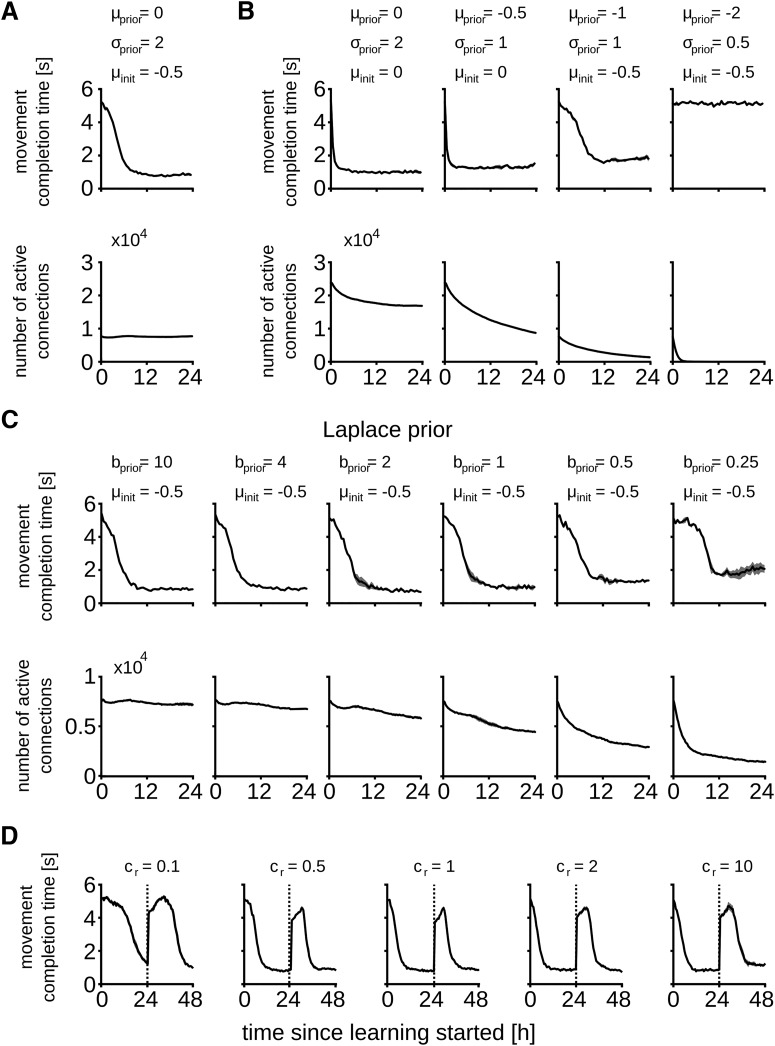
Impact of the prior distribution and reward amplitude on the synaptic dynamics. Task performance and total number of active synaptic connections throughout learning for different prior distributions and distribution of initial synaptic parameters. Synaptic parameters were initially drawn from a Gaussian distribution with mean *μ*_init_ and *σ* = 0.5. Comparison of the task performance and number of active synapses for the parameter set used in Figure 3 (***A***) and Gaussian prior distribution with different parameters (***B***). ***C***, In addition, a Laplace prior with different parameters was tested. The prior distribution and the initial synaptic parameters had a marked effect on the task performance and overall network connectivity. ***D***, Impact of the reward amplitude on the synaptic dynamics. Task performance is here measured for different values of *c_r_* to scale the amplitude of the reward signal. Dashed lines denote the task switch as in Figure 3.

Next, we investigated the role of the prior distribution and initial network configuration for the experiment in Figure 3. Figure 4 shows the performance and total number of active connections for different parameter settings. As in the previous experiments, we used in Figure 4*A*,*B* a Gaussian prior distribution with mean *μ* and variance *σ* ^2^. The preferred number of active connections changes with the prior, i.e., priors with smaller variance and low mean lead to sparser networks. Convergence to this preferred number can take >24 h depending on the initial connectivity. Different parameter settings can therefore lead to quite different network connectivities at a similar task performance. A too strong prior (e.g., *μ* = –2, *σ* = 0.5) leads to very sparse networks, thereby preventing learning.

In addition to the Gaussian prior distribution we tested a Laplace prior of the form $p_{S}\left(\theta_{i}\right)=1/2b\exp\left(-|\theta_{i}|/b\right),$ with zero mean and scale parameter *b* > 0 (Fig. 4*C*). This leads to a constant negative drift term in the parameter dynamics Equation 5, i.e., $\frac{\partial }{\partial \theta_{i}}\log p_{S}\left(\theta \right)=-1/b$ for active synaptic connections. A similar mechanism for synaptic weight decay was used previously (Rokni et al., 2007). Convergence to sparse connectivities is faster with this prior and good task performance can be reached by networks with less active connections compared to the Gaussian prior. For example, the network with *b* = 2 solved the task in 0.66 s on average using roughly 5700 active connections, whereas the best solution for the Gaussian prior was 0.83 s on average with typically >7500 active connections. Again, for the Laplace prior, parameters that enforced too sparse networks degraded task performance.

We next investigated whether a scaling of the amplitude of the reward signal *r*(*t*) while keeping the same prior has an influence on network performance. we introduced a scaling constant *c_r_* that can be used to modulate the amplitude of the reward signal (*c_r_* = 1 corresponds to the setting in Fig. 3; for details, see Materials and Methods). We repeated the experiment from Figure 3 (including the task change after 24 h) with *c_r_* ranging between 0.1 and 10. For values of *c_r_* smaller than 1 the effect of the second term of the synaptic dynamics (Eq. 5) is scaled down which results in an overall reduced learning speed and a stronger influence of the prior. Interestingly however, in all cases the network was able to compensate for the task change after 48 h of simulated biological time (see Fig. 4*D*, movement completion times of 983 ± 63, 894 ± 41, 820 ± 45, 743 ± 25, and 1181 ± 42 ms for *c_r_* = 0.1, 0.5, 1,5, and 10, respectively). In the next section we further investigate the role of the temperature *T* that controls the amount of noise in the synaptic dynamics.

### Relative contributions of spontaneous and activity-dependent synaptic processes

Dvorkin and Ziv (2016) analyzed the correlation of sizes of postsynaptic densities and spine volumes for synapses that shared the same pre- and postsynaptic neuron, called commonly innervated (CI) synapses, and also for synapses that shared in addition the same dendrite (CI_SD_). Activity-dependent rules for synaptic plasticity, such as Hebbian or STDP rules, on which previous models for network plasticity relied, suggest that the strength of CI and especially CI_SD_ synapses should be highly correlated. But both data from *ex vivo* (Kasthuri et al., 2015) and neural circuits in culture (Dvorkin and Ziv, 2016) show that postsynaptic density sizes and spine volumes of CI_SD_ synapses are only weakly correlated, with correlation coefficients between 0.23 and 0.34. Thus even with a conservative estimate that corrects for possible influences of their experimental procedure, >50% of the observed synaptic strength appears to result from activity-independent stochastic processes (Dvorkin and Ziv, 2016, their Fig. 8*E*); Bartol et al., (2015) had previously found larger correlations of synaptic strengths of CI_SD_ synapses for a smaller data set (based on 17 CI_SD_ pairs instead of the 72 pairs, 10 triplets, and two quadruplets in the *ex vivo* data from Kasthuri et al., 2015), but the spine volumes differed in these pairs also on average by a factor of around 2.

**Figure 5.**
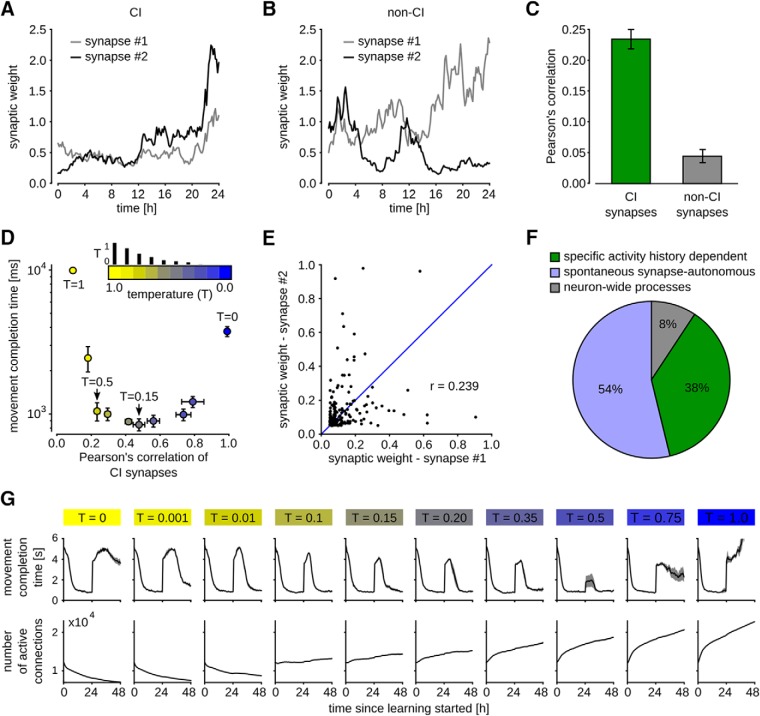
Contribution of spontaneous and neural activity-dependent processes to synaptic dynamics. ***A***, ***B***, Evolution of synaptic weights *w_i_* plotted against time for a pair of CI synapses in **a**, and non-CI synapses in ***B***, for temperature *T* = 0.5. ***C***, Pearson’s correlation coefficient computed between synaptic weights of CI and non-CI synapses of a network with *T* = 0.5 after 48 h of learning as in Figure 3*C*,*D*. CI synapses were only weakly, but significantly stronger correlated than non-CI synapses. ***D***, Impact of *T* on correlation of CI synapses (*x*-axis) and learning performance (*y*-axis). Each dot represents averaged data for one particular temperature value, indicated by the color. Values for *T* were 1.0, 0.75, 0.5, 0.35, 0.2, 0.15, 0.1, 0.01, 0.001, and 0.0. These values are proportional to the small vertical bars above the color bar. The performance (measured in movement completion time) is measured after 48 h for the learning experiment as in Figure 3*C*,*D*, where the network changed completely after 24 h. Good performance was achieved for a range of temperature values between 0.01 and 0.5. Too low (<0.01) or too high (>0.5) values impaired learning. Means ± SEM over five independent trials are shown. ***E***, Synaptic weights of 100 pairs of CI synapses that emerged from a run with *T* = 0.5. Pearson’s correlation is 0.239, comparable to the experimental data in Dvorkin and Ziv (2016), their Figure 8*A–D*. ***F***, Estimated contributions of activity history dependent (green), spontaneous synapse-autonomous (blue) and neuron-wide (gray) processes to the synaptic dynamics for a run with *T* = 0.15. The resulting fractions are very similar to those in the experimental data, see Dvorkin and Ziv (2016), their Figure 8*E*. ***G***, Evolution of learning performance and total number of active synaptic connections for different temperatures as in ***D***. Compensation for task perturbation was significantly faster with higher temperatures. Temperatures larger than 0.5 prevented compensation. Overall number of synapses was decreasing for temperatures *T* < 0.1 and increasing for *T* ≥ 0.1.

We asked how such a strong contribution of activity-independent synaptic dynamics affects network learning capabilities, such as the ones that were examined in Figure 3. We were able to carry out this test because many synaptic connections between neurons that were formed in our model consisted of more than one synapse. We classified pairs of synapses that had the same pre- and postsynaptic neuron as CI synapses (one could also call them CI_SD_ synapses, since the neuron model did not have different dendrites), and pairs with the same postsynaptic but different presynaptic neurons as non-CI synapses. Example traces of synaptic weights for CI and non-CI synapse pairs of our network model from Figure 3 are shown in Figure 5*A*,*B*. CI pairs were found to be more strongly correlated than non-CI pairs (Fig. 5*C*). However, also the correlation of CI pairs was quite low and varied with the temperature parameter *T* in Equation 5 (Fig. 5*D*). The correlation was measured in terms of the Pearson correlation (covariance of synapse pairs normalized between -1 and 1).

Since the correlation of CI pairs in our model depends on the temperate *T*, we analyzed the model of Figure 3 for different temperatures (the temperature had been fixed at *T* = 0.1 throughout the experiments for Fig. 3). In Figure 5*D*, the Pearson’s correlation coefficient for CI synapses is plotted together with the average performance achieved on the task of Figure 3*D–H* (24 h after the task switch) for networks with different temperatures *T*. The best performing temperature region for the task (0.01 ≤ *T* ≤ 0.5) roughly coincided with the region of experimentally measured values of Pearson’s correlation for CI synapses. Figure 5*E* shows the correlation of 100 CI synapse pairs that emerged from a run with *T* = 0.5. We found a value of *r* = 0.239 in this case. This value is in the order of the lowest experimentally found correlation coefficients in Dvorkin and Ziv (2016; both in culture and *ex vivo*, see their Figure 8*A–D*). The speed of compensation and the overall replenishing of synapses was strongly dependent on the temperature *T* (Fig. 5*G*). For *T* = 0, a complete compensation for the task changes was prevented (performance converged to 2.5 ± 0.2 s during a longer run of 96 h). The temperature region 0.01 ≤ *T* ≤ 0.5, which is consistent with experimentally measured Pearson’s correlation for CI synapses, leads to fastest task relearning, allowing for a compensation within ∼12 h of exposure. For *T* = 0.15, we found the best compensation capabilities and the closest match to experimentally measured correlations when the results of Dvorkin and Ziv (2016) were corrected for measurement limitations: a correlation coefficient of *r* = 0.46 ± 0.034 for CI synapses and 0.08 ±0.015 for non-CI synapse pairs (mean ± SEM over five trials, CI synapses were significantly stronger correlated than non-CI, *p* < 0.005 in all trials; statistical significance values based on two-tailed Mann–Whitney *U* test).

Dvorkin and Ziv (2016) further analyzed the ratio of contributions from different processes to the measured synaptic dynamics. They analyzed the contribution of neural activity history dependent processes, which amount for 36% of synapse dynamics in their data, and that of neuron-wide processes that were not specific to presynaptic activity, but specific to the activity of the postsynaptic neuron (8%). Spontaneous synapse-autonomous processes were found to explain 56% of the observed dynamics (see Dvorkin and Ziv, 2016, their Fig. 8*E*). The results from our model with *T* = 0.15, which are plotted in Figure 5*F*, match these experimentally found values quite well. Together, we found that the results of Dvorkin and Ziv (2016) are best explained by our model for a temperature parameter between *T* = 0.5 (corresponding to their lowest measured correlation coefficient) and *T* = 0.15 (corresponding to their most conservative estimate). This range of parameters coincided with well-functioning learning behavior in our model, which included a test of compensation capability for a change of the task after 24 h (Fig. 5*D*). Hence, our model suggests that a large component of stochastic synapse-autonomous processes, as it occurs in the data, supports efficient network learning and compensation for changes in the task.

## Discussion

Recent experimental data (Nagaoka et al., 2016; see also Dvorkin and Ziv, 2016, where in their Figure 8 also mouse brain data from Kasthuri et al., 2015 were reanalyzed) suggest that common models for learning in neural networks of the brain need to be revised, since synapses are subject to powerful processes that do not depend on pre- and postsynaptic neural activity. In addition, experimentally found network rewiring has so far not been integrated into models for reward-gated network plasticity. We have presented a theoretical framework that enables us to investigate and understand reward-based network rewiring and synaptic plasticity in the context of the experimentally found high level of activity-independent fluctuations of synaptic connectivity and synaptic strength. We have shown that the analysis of the stationary distribution of network configurations, in particular the Fokker–Planck equation from theoretical physics, allows us to understand how large numbers of local stochastic processes at different synapses can orchestrate global goal-directed network learning. This approach provides a new normative model for reward-gated network plasticity.

We have shown in Figure 2 that the resulting model is consistent with experimental data on dopamine-dependent spine dynamics reported in Yagishita et al. (2014) and that it provides an understanding how these local stochastic processes can produce function-oriented cortical-striatal connectivity. We have shown in Figure 3 that this model also elucidates reward-based self-organization of generic recurrent neural networks for a given computational task. We chose as benchmark task the production of a specific motor output in response to a cue, like in the experiments of Peters et al. (2014). Similarly as reported in Peters et al. (2014), the network connectivity and dynamics reorganized itself in our model, just driven by stochastic processes and rewards for successful task completion, and reached a high level of performance. Furthermore, it maintained this computational function despite continuously ongoing further rewiring and network plasticity. A quantitative analysis of the impact of stochasticity on this process has shown in Figure 5 that the network learns best when the component of synaptic plasticity that does not depend on neural activity is fairly large, as large as reported in the experimental data of Kasthuri et al. (2015); Dvorkin and Ziv (2016).

Our approach is based on experimental data for the biological implementation level of network plasticity, i.e., for the lowest level of the Marr hierarchy of models (Marr and Poggio, 1976). However, we have shown that these experimental data have significant implications for understanding network plasticity on the top level (“what is the functional goal?”) and the intermediate algorithmic level (“what is the underlying algorithm?”) of the Marr hierarchy. They suggest for the top level that the goal of network plasticity is to evaluate a posterior distribution of network configurations. This posterior integrates functional demands formalized by the expected discounted reward V(θ) with a prior $p_{S}(\text{θ})$ in a multiplicative manner $p^{*}\left(\theta \right)\propto p_{S}\left(\theta \right)\times V\left(\theta \right)$. Priors can represent structural constraints as well as results of preceding learning experiences and innate programs. Since our model samples from a distribution proportional to $p^{*}{\left(\text{θ}\right)}^{1/T}$, for *T* = 1, our model suggests to view reward-gated network plasticity as Bayesian inference over network configurations on a slow time scale (for details, see Materials and Methods, Probabilistic framework for reward-modulated learning). For a temperature parameter *T* ≠ 1, the model samples from a tempered version of the posterior, which generalizes the basic Bayesian approach. This Bayesian perspective also creates a link to previous work on Bayesian reinforcement learning (Vlassis et al., 2012; Rawlik et al., 2013). We note however that we do not consider parameter adaptation in our framework to implement full Bayesian learning, as there is no integration over the posterior parameter settings to obtain network outputs (or actions in a reinforcement learning context). Even if one would do that, it would be of little practical use, since the sampling would be much too slow in any but the simplest networks. The experimental data suggest for the intermediate algorithmic level of the Marr hierarchy a strong reliance on stochastic search (“synaptic sampling”). The essence of the resulting model for reward-gated network learning is illustrated in Figure 1. The traditional view of deterministic gradient ascent (policy gradient) in the landscape (Fig. 1*B*) of reward expectation is first modified through the integration of a prior (Fig. 1*C*), and then through the replacement of gradient ascent by continuously ongoing stochastic sampling (policy sampling) from the posterior distribution of Figure 1*D*, which is illustrated in Figure 1*E*,*F*.

This model explains a number of experimental data that had not been addressed by previous models. Continuously ongoing stochastic sampling of network configurations suggests that synaptic connectivity does not converge to a fixed point solution but rather undergoes permanent modifications (Fig. 3*H*,*I*). This behavior is compatible with reports of continuously ongoing spine dynamics and axonal sprouting even in the adult brain (Holtmaat et al., 2005; Stettler et al., 2006; Yasumatsu et al., 2008; Holtmaat and Svoboda, 2009; Yamahachi et al., 2009; Loewenstein et al., 2011, 2015). Recently proposed models to maintain stable network function in the presence of highly volatile spine dynamics suggest that subsets of connections are selectively stabilized to support network function (Berry and Nedivi, 2017; Mongillo et al., 2017). Our result shows that high task performance can be reached in spiking neural networks in the presence of high volatility of all synapses. Still our model can be extended with a process that selectively stabilizes synapses on longer timescales as demonstrated in Figure 3*F*. In addition, our model predicts that not only synaptic spine dynamics but also changes of synaptic efficacies show a large stochastic component on all timescales.

The continuously ongoing parameter changes induce continuously ongoing changes in the assembly sequences that accompany and control a motor response (Fig. 3*D*). These changes do not impair the performance of the network, but rather enable the network to explore different but equally good solutions when exposed for many hours to the same task (Fig. 3*I*). Such continuously ongoing drifts of neural codes in functionally less relevant dimensions have already been observed experimentally in some brain areas (Ziv et al., 2013; Driscoll et al., 2017). Our model also suggests that the same computational function is realized by the same neural circuit in different individuals with drastically different parameters, a feature which has already been addressed (Prinz et al., 2004; Grashow et al., 2010; Tang et al., 2010; Marder, 2011). In fact, this degeneracy of neural circuits is thought to be an important property of biological neural networks (Prinz et al., 2004; Marder and Goaillard, 2006; Marder, 2011). Our model networks automatically compensate for disturbances by moving their continuously ongoing sampling of network configurations to a new region of the parameter space, as illustrated by the response to the disturbance marked by an asterisk in Figure 3*I*.

Our theoretical framework is consistent with experimental data that showed drifts of neural representations in motor learning (Rokni et al., 2007). In that article, a stochastic plasticity model was proposed that is structurally similar to our model. It was shown in computer simulations that a simple feed forward rate-based neural network is able to retain stable functionality despite of such stochastic parameter changes. The authors hypothesized that this is the case because network parameters move on a submanifold in parameter space with constant performance. Our theoretical framework provides a mathematical justification for their hypothesis in general, but also refines these statements. It shows that the network samples network configurations (including the rewiring of connections that was not considered in Rokni et al., 2007) from a well-defined distribution. The manifold that is visited during the learning process is given by the high-probability regions of this distribution, but in principle, also suboptimal regions could be visited. Such suboptimal regions are however highly unlikely if the parameter space is overcomplete, i.e., if large volumes of the parameter space lead to good performance. Hence, in comparison with Rokni et al. (2007), this work provides the following features: (1) it provides a quantitative mathematical framework for the qualitative descriptions in Rokni et al. (2007) that allows a rigorous understanding of the plasticity processes; (2) it includes synaptic rewiring, reproducing experimental data on this topic and providing a hypothesis on its computational role; and (3), it is able to tackle the case of recurrent spiking neural networks as compared to feed forward rate models.

We have shown in Figure 3*F* that despite these permanent parameter drifts, the task performance in our model remains stable for many simulated days if reward delivery is stopped. At the same time, the model is also able to continuously adapt to changes in the task (Fig. 3*C–E*). These results suggest that our model keeps a quite good balance between stability and plasticity (Abraham and Robins, 2005), which has been shown previously to be one important functional aspect of network rewiring (Fauth et al., 2015). Furthermore, we have shown in Figure 3*F* that the structural priors over synaptic parameters can be used to stabilize synaptic parameters similar to previous models of synaptic consolidation (Fusi et al., 2005; Kirkpatrick et al., 2017). In addition, more complex prior distributions over multiple synapses could be used to model homeostatic processes and clustering of synapses. The latter has been suggested as a mechanism to tackle the stability-plasticity dilemma (Fares and Stepanyants, 2009).

In conclusion the mathematical framework presented in this article provides a principled way of understanding the complex interplay of deterministic and stochastic processes that underlie the implementation of goal-directed learning in neural circuits of the brain. It also offers a solution to the problem how reliable network computations can be achieved with a dynamic connectome (Rumpel and Triesch, 2016). We have argued that the stationary distribution of the high-dimensional parameter vector θ that results from large numbers of local stochastic processes at the synapses provides a time-invariant perspective of salient properties of a network. Standard reward-gated plasticity rules can achieve that this stationary distribution has most of its mass on regions in the parameter space that provide good network performance. The stochastic component of synaptic dynamics can flatten or sharpen the resulting stationary distribution, depending on whether the scaling parameter *T* (temperature) of the stochastic component is larger or smaller than 1. A functional benefit of this stochastic component is that the network keeps exploring its parameter space even after a well-performing region has been found, providing one mechanism to tackle the exploration-exploitation dilemma (Fig. 2*J*). This enables the network to migrate quickly and automatically to a better performing region when the network or task changes. We found in the case of the motor learning task of Figure 3 that a temperature *T* around 0.15, which lies in the same range as related experimental data (Fig. 5*D*), suffices to provide this functionally important compensation capability. The same mathematical framework can also be applied to artificial neural networks, leading to a novel brain-inspired learning algorithm that uses rewiring to train deep networks under the constraint of very sparse connectivity (Bellec et al., 2017).

## Materials and Methods

### Probabilistic framework for reward-modulated learning

The classical goal of reinforcement learning is to maximize the expected future discounted reward V(θ) given by

(6) $$V\left(\theta \right)={\left\langle \int_{0}^{\infty }e^{-\frac{\tau }{\tau_{e}}}r\left(\tau \right)d\tau \right\rangle }_{p\left(r|\theta \right)}.$$

In Equation 6, we integrate over all future rewards *r*(*τ*), while discounting more remote rewards exponentially with a discount rate *τ_e_*, which for simplicity was set equal to 1 s in this paper. We find (Eq. 15) that this time constant *τ_e_* is immediately related to the experimentally studied time window or eligibility trace for the influence of dopamine on synaptic plasticity (Yagishita et al., 2014). This property is true in general for reward-based learning rules that make use of eligibility traces and is not unique to our model. The expectation in Equation 6 is taken with respect to the distribution p(r|θ) over sequences r={r(τ),τ≥0} of future rewards that result from the given set of synaptic parameters θ. The stochasticity of the reward sequence r arises from stochastic network inputs, stochastic network responses, and stochastic reward delivery. The resulting distribution p(r|θ) of reward sequences r for the given parameters θ can also include influences of network initial conditions by assuming some distribution over these initial conditions. Network initial conditions include for example initial values of neuron membrane voltages and refractory states of neurons. The role of initial conditions on network learning is further discussed below when we consider the online learning scenario (see Reward-modulated synaptic plasticity approximates gradient ascent on the expected discounted reward).

There exists a close relationship between reinforcement learning and Bayesian inference (Botvinick and Toussaint, 2012; Vlassis et al., 2012; Rawlik et al., 2013). To make this relationship apparent, we define our model for reward-gated network plasticity by introducing a binary random variable *v_b_* that represents the currently expected future discounted reward in a probabilistic manner. The likelihood $p_{N}\left(v_{b}=1|\text{θ}\right)$ is determined in this theoretical framework by the expected future discounted reward Equation 6 that is achieved by a network with parameter set θ (Rawlik et al., 2013):

(7) $$p_{N}\left(v_{b}=1|\text{θ}\right)\;\equiv \;\frac{1}{Z_{V}}V(\text{θ})\;,$$

where $Z_{V}$ denotes a constant, that assures that Equation 7 is a correctly normalized probability distribution. Thus reward-based network optimization can be formalized as maximizing the likelihood $p_{N}\left(v_{b}=1|\text{θ}\right)$ with respect to the network configuration θ. Structural constraints can be integrated into a stochastic model for network plasticity through a prior $p_{S}(\text{θ})$ over network configurations. Hence reward-gated network optimization amounts from a theoretical perspective to learning of the posterior distribution $p^{*}(\text{θ}|v_{b}=1)$, which by Bayes’ rule is defined (up to normalization) by $p_{S}\left(\text{θ}\right)\cdot p_{N}\left(v_{b}=1|\text{θ}\right)$. Therefore, the learning goal can be formalized in a compact form as evaluating the posterior distribution $p^{*}(\text{θ}|v_{b}=1)$ of network parameters θ under the constraint that the abstract learning goal *v_b_* = 1 is achieved.

More generally, one is often interested in a tempered version of the posterior

(8) $$p_{T}^{*}(\text{θ})\equiv \frac{1}{Z}p^{*}{\left(\text{θ}|v_{b}=1\right)}^{\frac{1}{T}}\;,$$

where Z is a suitable normalization constant and *T* > 0 is the temperature parameter that controls the “sharpness” of $p_{T}^{*}(\text{θ})$. For *T* = 1, $p_{T}^{*}(\text{θ})$ is given by the original posterior, *T* < 1 emphasizes parameter values with high probability in the posterior, while *T* > 1 leads to parameter distributions $p_{T}^{*}(\text{θ})$ which are more uniformly distributed than the posterior.

### Analysis of policy sampling

Here, we prove that the stochastic parameter dynamics Equation 5 samples from the tempered posterior distribution $p_{T}^{*}(\text{θ})$ given in Equation 8. In Results, we suppressed time-dependencies to simplify notation. We reiterate Equation 3 with explicit time-dependencies of parameters:

(9) $${d\theta_{i}\left(t\right)=\beta \frac{\partial }{\partial \theta_{i}}\log p^{*}\left(\theta |v_{b}=1\right)|}_{\theta \left(t\right)}dt+\sqrt{2\beta T}dW_{i},$$

where the notation ${\frac{\partial }{\partial \theta_{i}}f\left(\theta \right)|}_{\theta \left(t\right)}$ denotes the derivative of f(θ) with respect to *θ_i_* evaluated at the current parameter values θ(t). By Bayes’ rule, the derivative of the log posterior is the sum of the derivatives of the log prior and the log likelihood:

$$\frac{\partial }{\partial \theta_{i}}\log p^{*}(\text{θ}|v_{b}=1)\;=\;\frac{\partial }{\partial \theta_{i}}\log p_{S}\left(\text{θ}\right)+\frac{\partial }{\partial \theta_{i}}\log p_{N}\left(v_{b}=1|\text{θ}\right)\;=\;\frac{\partial }{\partial \theta_{i}}\log p_{S}\left(\text{θ}\right)+\frac{\partial }{\partial \theta_{i}}\log V(\text{θ})\;,$$

which allows us to rewrite Equation 9 as

(10) $$d\theta_{i}\left(t\right)=\beta \left({\frac{\partial }{\partial \theta_{i}}\log p_{S}\left(\theta \right)|}_{\theta \left(t\right)}+{\frac{\partial }{\partial \theta_{i}}\log V\left(\theta \right)|}_{\theta \left(t\right)}\right)dt+\sqrt{2\beta T}dW_{i},$$

which is identical to the form of Equation 5, where the contributions of $p_{S}\left(\text{θ}\right)$ and V(θ) are given explicitly.

The fundamental property of the synaptic sampling dynamics Equation 9 is formalized in Theorem 1 and proven below. Before we state the theorem, we briefly discuss its statement in simple terms. Consider some initial parameter setting θ(0). Over time, the parameters change according to the dynamics (9). Since the dynamics include a noise term, the exact value of the parameters θ(t) at some time *t* > 0 cannot be determined. However, it is possible to describe the exact distribution of parameters for each time *t*. We denote this distribution by $p_{\text{FP}}(\text{θ},t)$, where the FP subscript stands for Fokker–Planck, since the evolution of this distribution is described by the Fokker–Planck equation (Eq. 11) given below. Note that we make the dependence of this distribution on time explicit in this notation. It can be shown that for the dynamics of Equation 11, $p_{\text{FP}}(\text{θ},t)$ converges to a well-defined and unique stationary distribution in the limit of large *t*. Of practical relevance is the so-called burn-in time after which the distribution of parameters is very close to the stationary distribution. Note that the parameters will continue to change. Nevertheless, at any time *t* after the burn in, we can expect the parameter vector θ(t) to be situated at a particular value with the probability (density) given by the stationary distribution (Fig. 1*D*,*F*). Any distribution that is invariant under the parameter dynamics is a stationary distribution. Here, invariance means: when one starts with an invariant distribution over parameters in the Fokker–Planck equation, the dynamics are such that this distribution will be kept forever (we will use this below in the proof of Theorem 1). Theorem 1 states that the parameter dynamics leaves $p_{T}^{*}(\text{θ})$ given in Equation 8 invariant, i.e., it is a stationary distribution of the network parameters. Note that in general, the stationary distribution may not be uniquely defined. That is, it could happen that for two different initial parameter values, the network reaches two different stationary distributions. Theorem 1 further states that for the synaptic sampling dynamics, the stationary distribution is unique, i.e., the distribution $p_{T}^{*}(\text{θ})$ is reached from *any* initial parameter setting when the conditions of the theorem apply. We now state Theorem 1 formally. To simplify notation, we drop in the following the explicit time dependence of the synaptic parameters θ.

Theorem 1. Let $p^{*}\left(\theta |v_{b}=1\right)$ be a strictly positive, continuous probability distribution over parameters θ, twice continuously differentiable with respect to θ, and let β > 0. Then the set of stochastic differential Equation 9 leaves the distribution $p_{T}^{*}(\text{θ})$ (8) invariant. Furthermore, $p_{T}^{*}(\text{θ})$ is the unique stationary distribution of the sampling dynamics. The proof is analogous to the one provided in Kappel et al. (2015). The stochastic differential equation Equation 9 translates into a Fokker–Planck equation (Gardiner, 2004) that describes the evolution of the distribution over parameters θ

(11) $$\frac{\partial }{\partial t}p_{\text{FP}}\left(\theta ,t\right)=\underset{i}{\sum }-\frac{\partial }{\partial \theta_{i}}\left(\beta \frac{\partial }{\partial \theta_{i}}\log p^{*}\left(\theta |v_{b}=1\right)\right)p_{\text{FP}}\left(\theta ,t\right)+\frac{\partial^{2}}{\partial \theta_{i}^{2}}\left(\beta Tp_{\text{FP}}(\theta ,t)\right),$$

where $p_{\text{FP}}(\text{θ},t)$ denotes the distribution over network parameters at time *t*. To show that $p_{T}^{*}(\text{θ})$ leaves the distribution invariant, we have to show that $\frac{\partial }{\partial t}p_{\text{FP}}(\text{θ},t)=0$ (i.e., $p_{\text{FP}}(\text{θ},t)$ does not change) if we set $p_{\text{FP}}(\text{θ},t)$ to $p_{T}^{*}(\text{θ})$ on the right hand-side of Equation 11. Plugging in the presumed stationary distribution $p_{T}^{*}(\text{θ})$ for $p_{\text{FP}}(\text{θ},t)$ on the right hand-side of Equation 11, one obtains

$$\begin{array}{c} \frac{\partial }{\partial t}p_{\text{FP}}\left(\theta ,t\right)=\underset{i}{\sum }-\frac{\partial }{\partial \theta_{i}}\left(\beta \frac{\partial }{\partial \theta_{i}}\log p^{*}\left(\theta |v_{b}=1\right)p_{T}^{*}\left(\theta \right)\right)+\frac{\partial^{2}}{\partial \theta_{i}^{2}}\left(\beta Tp_{T}^{*}\left(\theta \right)\right)\\ =\underset{i}{\sum }-\frac{\partial }{\partial \theta_{i}}\left(\beta p_{T}^{*}\left(\theta \right)\frac{\partial }{\partial \theta_{i}}\log p^{*}\left(\theta |v_{b}=1\right)\right)+\frac{\partial }{\partial \theta_{i}}\left(\beta T\frac{\partial }{\partial \theta_{i}}p_{T}^{*}\left(\theta \right)\right)\\ =\underset{i}{\sum }-\frac{\partial }{\partial \theta_{i}}\left(\beta p_{T}^{*}\left(\theta \right)\frac{\partial }{\partial \theta_{i}}\log p^{*}\left(\theta |v_{b}=1\right)\right)+\frac{\partial }{\partial \theta_{i}}\left(\beta Tp_{T}^{*}\left(\theta \right)\frac{\partial }{\partial \theta_{i}}\log p_{T}^{*}\left(\theta \right)\right), \end{array}$$

which by inserting $p_{T}^{*}\left(\theta \right)=\frac{1}{Z}p^{*}{\left(\theta |v_{b}=1\right)}^{1/T}$, with normalizing constant Z, becomes

$$\frac{\partial }{\partial t}p_{FP}\left(\theta ,t\right)=\frac{1}{Z}\underset{i}{\sum }-\frac{\partial }{\partial \theta_{i}}\left(\beta p^{*}\left(\theta \right)\frac{\partial }{\partial \theta_{i}}\log p^{*}\left(\theta |v_{b}=1\right)\right)+\frac{\partial }{\partial \theta_{i}}\left(\beta Tp^{*}\left(\theta \right)\frac{1}{T}\frac{\partial }{\partial \theta_{i}}\log p^{*}\left(\theta |v_{b}=1\right)\right)=\underset{i}{\sum }0=0.$$

This proves that $p_{T}^{*}(\text{θ})$ is a stationary distribution of the parameter sampling dynamics Equation 9. Since *β* is strictly positive, this stationary distribution is also unique (see Section 3.7.2 in Gardiner, 2004).

The unique stationary distribution of Equation 11 is given by $p_{T}^{*}(\text{θ})=\frac{1}{Z}p^{*}{\left(\text{θ}|v_{b}=1\right)}^{\frac{1}{T}}$, i.e., $p_{T}^{*}(\text{θ})$ is the only solution for which $\frac{\partial }{\partial t}p_{\text{FP}}(\text{θ},t)$ becomes 0, which completes the proof.

The uniqueness of the stationary distribution follows because each parameter setting can be reached from any other parameter setting with non-zero probability (ergodicity). The stochastic process can therefore not get trapped in cycles or absorbed into a subregion of the parameter space. The time spent in a certain region of the parameter space is therefore directly proportional to the probability of that parameter region under the posterior distribution. The proof requires that the posterior distribution is smooth and differentiable with respect to the synaptic parameters. This is not true in general for a spiking neural network. In our simulations we used a stochastic neuron model (defined in the next section). As the reward landscape in our case is defined by the expected discounted reward (Reward-modulated synaptic plasticity approximates gradient ascent on the expected discounted reward), a probabilistic network tends to smoothen this landscape and therefore the posterior distribution.

### Network model

Plasticity rules for this general framework were derived based on a specific spiking neural network model, which we describe in the following. All reported computer simulations were performed with this network model. We considered a general network scaffold N of *K* neurons with potentially asymmetric recurrent connections. Neurons are indexed in an arbitrary order by integers between 1 and *K*. We denote the output spike train of a neuron *k* by *z_k_*(*t*). It is defined as the sum of Dirac delta pulses positioned at the spike times $t_{k}^{\left(1\right)},t_{k}^{\left(2\right)},\ldots$, i.e., $z_{k}(t)=\sum_{l}\delta (t-t_{k}^{\left(l\right)})$. Potential synaptic connections are also indexed in an arbitrary order by integers between 1 and *K*_syn,_ where *K*_syn_ denotes the number of potential synaptic connections in the network. We denote by pre*_i_* and post*_i_* the index of the pre- and postsynaptic neuron of synapse *i*, respectively, which unambiguously specifies the connectivity in the network. Further, we define syn*_k_* to be the index set of synapses that project to neuron *k*. Note that this indexing scheme allows us to include multiple (potential) synaptic connections between a given pair of neurons. We included this experimentally observed feature of biological neuronal networks in all our simulations. We denote by *w_i_*(*t*) the synaptic efficacy of the *i*-th synapse in the network at time *t*.

Network neurons were modeled by a standard stochastic variant of the spike response model (Gerstner et al., 2014). In this model, the membrane potential of a neuron *k* at time *t* is given by

(12) $$u_{k}\left(t\right)=\underset{i\in {\text{SYN}}_{k}}{\sum }y_{{\text{PRE}}_{i}}\left(t\right)w_{i}\left(t\right)+\vartheta_{k}\left(t\right),$$

where $\vartheta_{k}(t)$ denotes the slowly changing bias potential of neuron *k*, and $y_{pre_{i}}(t)$ denotes the trace of the (unweighted) postsynaptic potentials (PSPs) that neuron pre*_i_* leaves in its postsynaptic synapses at time *t*. More precisely, it is defined as $y_{pre_{i}}(t)=z_{pre_{i}}(t)*\epsilon (t)$ given by spike trains filtered with a PSP kernel of the form $\epsilon \left(t\right)=\Theta \left(t\right)\frac{\tau_{r}}{\tau_{m}-\tau_{r}}\left(e^{-t/\tau_{m}}-e^{-t/\tau_{r}}\right)$, with time constants *τ_m_* = 20 ms and *τ_r_* = 2 ms, if not stated otherwise. Here * denotes convolution and Θ(⋅) is the Heaviside step function, i.e., Θ(*x*) = 1 for *x* ≥ 0 and 0 otherwise.

The synaptic weights *w_i_*(*t*) in Equation 12 were determined by the synaptic parameters *θ_i_*(*t*) through the mapping Equation 1 for *θ_i_*(*t*) > 0. Synaptic connections with *θ_i_*(*t*) ≤ 0 were interpreted as not functional (disconnected) and *w_i_*(*t*) was therefore set to 0 in that case.

The bias potential $\vartheta_{k}(t)$ in Equation 12 implements a slow adaptation mechanism of the intrinsic excitability, which ensures that the output rate of each neuron stays near the firing threshold and the neuron maintains responsiveness (Desai et al., 1999; Fan et al., 2005). We used a simple adaptation mechanism which was updated according to

(13) $$\tau_{\vartheta }\frac{d\vartheta_{k}\left(t\right)}{dt}=\nu_{0}-z_{k}\left(t\right),$$

where $\tau_{\vartheta }=50\text{s}$ is the time constant of the adaptation mechanism and *ν*_0_ = 5 Hz is the desired output rate of the neuron. In our simulations, the bias potential $\vartheta_{k}(t)$ was initialized at -3 and then followed the dynamics given in Equation 13. We found that this regularization significantly increased the performance and learning speed of our network model. In Remme and Wadman (2012), a similar mechanism was proposed to balance activity in networks of excitatory and inhibitory neurons. The regularization used here can be seen as a simplified version of this mechanism that regulates the mean firing rate of each excitatory neuron using a simple linear control loop and thereby stabilizes the output behavior of the network.

We used a simple refractory mechanism for our neuron model. The firing rate, or intensity, of neuron *k* at time *t* is defined by the function $f_{k}(t)\;=\;f(u_{k}(t),\rho_{k}(t))$, where *ρ_k_*(*t*) denotes a refractory variable that measures the time elapsed since the last spike of neuron *k*. We used an exponential dependence between membrane potential and firing rate, such that the instantaneous firing rate of the neuron *k* at time *t* can be written as

(14) $$f_{k}(t)\;=\;f(u_{k},\rho_{k})\;=\;\exp(u_{k})\Theta (\rho_{k}-t_{\text{ref}})\;.$$

Furthermore, we denote by $f_{post_{i}}(t)$ the firing rate of the neuron postsynaptic to synapse *i*. If not stated otherwise we set the refractory time *t*_ref_ to 5 ms. In addition, a subset of neurons was clamped to some given firing rates (input neurons), such that *f_k_*(*t*) of these input neurons was given by an arbitrary function. We denote the spike train from these neurons by x(t), the network input.

### Synaptic dynamics for the reward-based synaptic sampling model

Here, we provide additional details on how the synaptic parameter dynamics Equation 5 was computed. We will first provide an intuitive interpretation of the equations and then provide a detailed derivation in the next section. The second term $\frac{\partial }{\partial \theta_{i}}\log V(\text{θ})$ of Equation 5 denotes the gradient of the expected future discounted reward Equation 6. In general, optimizing this function has to account for the case where rewards are provided after some delay period. It is well known that this distal reward problem can be solved using plasticity mechanisms that make use of eligibility traces in the synapses that are triggered by near coincident spike patterns, but their consolidation into the synaptic weights is delayed and gated by the reward signal *r*(*t*) (Sutton and Barto, 1998; Izhikevich, 2007). The theoretically optimal shape for these eligibility traces can be derived using the reinforcement learning theory and depends on the choice of network model. For the spiking neural network model described above, the gradient $\frac{\partial }{\partial \theta_{i}}\log V(\text{θ})$ can be estimated through a plasticity mechanism that uses an eligibility trace *e_i_*(*t*) in each synapse *i* which gets updated according to

(15) $$\frac{de_{i}\left(t\right)}{dt}=-\frac{1}{\tau_{e}}e_{i}\left(t\right)+w_{i}\left(t\right)y_{{\text{PRE}}_{i}}\left(t\right)\left(z_{{\text{POST}}_{i}}\left(t\right)-f_{{\text{POST}}_{i}}\left(t\right)\right),$$

where *τ_e_* = 1 s is the time constant of the eligibility trace. Recall that pre*_i_* denotes the index of the presynaptic neuron and post*_i_* the index of the postsynaptic neuron for synapse *i*. In Equation 15, $z_{post_{i}}(t)$ denotes the postsynaptic spike train, $f_{post_{i}}(t)$ denotes the instantaneous firing rate (Eq. 14) of the postsynaptic neuron and $w_{i}\left(t\right)y_{{\text{PRE}}_{i}}\left(t\right)$ denotes the PSP under synapse *i*.

The last term of Equation 15 shares salient properties with standard STDP learning rules, since plasticity is enabled by the presynaptic term $y_{pre_{i}}(t)$ and gated by the postsynaptic term $\left(z_{post_{i}}(t)-f_{post_{i}}(t)\right)$ (Pfister et al., 2006). The latter term also regularizes the plasticity mechanism such that synapses stop growing if the firing probability $f_{post_{i}}(t)$ of the postsynaptic neuron is already close to one.

The eligibility trace Equation 15 is multiplied by the reward *r*(*t*) and integrated in each synapse *i* using a second dynamic variable

(16) dgi(t)dt=−1τggi(t)+(r(t)r^(t)+α)ei(t),

where $\hat{r}(t)$ is a low-pass filtered version of *r*(*t*) (Reward-modulated synaptic plasticity approximates gradient ascent on the expected discounted reward). The variable *g_i_*(*t*) combines the eligibility trace and the reward, and averages over the time scale *τ_g_*. *α* is a constant offset on the reward signal. This parameter can be set to an arbitrary value without changing the stationary dynamics of the model (see next section). In our simulations, this offset *α* was chosen slightly above 0 (*α* = 0.02) such that small parameter changes were also present without any reward, as observed previously (Yagishita et al., 2014). Furthermore, *α* does not have to be chosen constant. E.g., this term can be used to incorporate predictions about the reward outcome by setting *α* to the negative of output of a critic network that learns to predict future reward. This approach has been previously studied in Frémaux et al. (2013) to model experimental data of Schultz et al. (1997); Schultz (2002). In the experiment (Fig. 4*D*), we included in Equation 16 the scaling constant *c_r_* to modulate the reward term $\left(\frac{r(t)}{\hat{r}(t)}+\alpha \right)$.

In the next section, we show that *g_i_*(*t*) approximates the gradient of the expected future reward with respect to the synaptic parameter. In our simulations we found that incorporating the low-pass filtered eligibility traces (Eq. 16) into the synaptic parameters works significantly better than using the eligibility traces directly for weight updates, although the latter approach was taken in a number of previous studies (Pfister et al., 2006; Legenstein et al., 2008; Urbanczik and Senn, 2009). Equation 16 essentially combines the eligibility trace with the reward and smoothens the resulting trace with a low-pass filter with time constant *τ_g_*. This time constant has been chosen to be in the order of spontaneous decay of disinhibited CaMKII in the synapse which is closely related to spine enlargement in the dopamine-gated STDP protocol of Yagishita et al., 2014 (compare their Figs. 3*F*, 4*C*).

$\hat{r}(t)$ in Equation 16 is a low-pass filtered version of *r*(*t*) that scales the synaptic updates. It was implemented through $\tau_{a}\frac{d\hat{r}(t)}{dt}=-\hat{r}(t)+r(t)$, with *τ_a_* = 50 s. The value of *τ_a_* has been chosen to equal *τ_g_* based on theoretical considerations (see below, Online learning). This scaling of the reward signal has the following effect. If the current reward *r*(*t*) exceeds the average reward $\hat{r}(t)$, the effect of the neuromodulatory signal *r*(*t*) will be >1. On the other hand, if the current reward is below average synaptic updates will be weighted by a term significantly lower than 1. Therefore, parameter updates are preferred for which the current reward signal exceeds the average.

Similar plasticity rules with eligibility traces in spiking neural networks have previously been proposed by several authors (Seung, 2003; Xie and Seung, 2004; Pfister et al., 2006; Florian, 2007; Izhikevich, 2007; Legenstein et al., 2008; Urbanczik and Senn, 2009; Frémaux et al., 2010, 2013). In Frémaux et al. (2013), also a method to estimate the neural firing rate $f_{post_{i}}(t)$ from back-propagating action potentials in the synapses has been proposed. The main difference to these previous approaches is that the activity-dependent last term in Equation 15 is scaled by the current synaptic weight *w_i_*(*t*). This weight-dependence of the update equations induces multiplicative synaptic dynamics and is a consequence of the exponential mapping Equation 1 (see derivation in the next section). This is an important property for a network model that includes rewiring. Note, that for retracted synapses (*w_i_*(*t*) = 0), both *e_i_*(*t*) and *g_i_*(*t*) decay to zero (within few minutes in our simulations). Therefore, we find that the dynamics of retracted synapses is only driven by the first (prior) and last (random fluctuations) term of Equation 5 and are independent from the network activity. Thus, retracted synapses spontaneously reappear also in the absence of reward after a random amount of time.

The first term in Equation 5 is the gradient of the prior distribution. We used a prior distribution that pulls the synaptic parameters toward *θ_i_*(*t*) = 0 such that unused synapses tend to disappear and new synapses are permanently formed. If not stated otherwise we used independent Gaussian priors for the synaptic parameters

$$p_{S}\left(\theta \right)=\underset{i}{\prod }p_{S}\left(\theta_{i}\left(t\right)\right),\;\text{with}\;p_{S}\left(\theta_{i}\left(t\right)\right)=\frac{1}{\sigma \sqrt{2\pi }}\exp\left(-\frac{{\left(\theta_{i}\left(t\right)-\mu \right)}^{2}}{2\sigma^{2}}\right),$$

where *σ* is the standard deviation of the prior distribution. Using this, we find that the contribution of the before the online parameter update equation is given by

(17) $$\frac{\partial }{\partial \theta_{i}}\log p_{S}\left(\theta \right)=\frac{1}{\sigma^{2}}\left(\mu -\theta_{i}\left(t\right)\right).$$

Finally, by plugging Equations 17, 16 into Equation 5, the synaptic parameter changes at time *t* are given by

(18) $$d\theta_{i}\left(t\right)=\beta \left(\frac{1}{\sigma^{2}}\left(\mu -\theta_{i}\left(t\right)\right)+g_{i}\left(t\right)\right)dt+\sqrt{2\beta T}dW_{i}.$$

We tuned the parameters of the prior distribution by hand to achieve good results on the task presented in Figure 3 (for a comparison of different prior distributions, see Fig. 4). These parameters were given by *σ* = 2 and *μ* = 0 and were used throughout all experiments if not stated otherwise. By inspecting Equation 18, it becomes immediately clear that the parameter dynamics follow an Ornstein–Uhlenbeck process if the activity-dependent second term is inactive (in the absence of reward), i.e., if *g_i_*(*t*) = 0. In this case, the dynamics are given by the deterministic drift toward the mean value *μ* and the stochastic diffusion fueled by the Wiener process $W_{i}$. The temperature *T* and the standard deviation *σ* scale the contribution of these two forces.

### Reward-modulated synaptic plasticity approximates gradient ascent on the expected discounted reward

We first consider a theoretical setup where the network is operated in arbitrarily long episodes such that in each episode a reward sequence r is encountered. The reward sequence r can be any discrete or real-valued function that is positive and bounded. The episodic scenario is useful to derive exact batch parameter update rules, from which we will then deduce online learning rules. Due to stochastic network inputs, stochastic network responses, and stochastic reward delivery, the reward sequence r is stochastic.

The classical goal of reinforcement learning is to maximize the function V(θ) of discounted expected rewards Equation 6. Policy gradient algorithms perform gradient ascent on V(θ) by changing each parameter *θ_i_* in the direction of the gradient $\partial \log V(\text{θ})/\partial \theta_{i}$. Here, we show that the parameter dynamics Equations 15, 16 approximate this gradient, i.e., $g_{i}(t)\approx \partial \log V(\text{θ})/\partial \theta_{i}$.

It is natural to assume that the reward signal *r*(*τ*) only depends indirectly on the parameters θ, through the history of network spikes *z_k_*(*τ*) up to time *τ*, which we write as $z\left(\tau \right)=\left\{z_{k}\left(s\right)|0\leq s<\tau ,1\leq k\leq K\right\},$ i.e., $p_{N}\left(r(t),z(t)|\text{θ}\right)=p\left(r(t)|z(t)\right)p_{N}\left(z(t)|\text{θ}\right)$. We can first expand the expectation ${〈\cdot 〉}_{p(\text{r}|\text{θ})}$ in Equation 6 to be taken over the joint distribution p(r,z|θ) over reward sequences r and network trajectories **z**. The derivative

(19) $$\frac{\partial }{\partial \theta_{i}}\log V\left(\theta \right)=\frac{1}{V\left(\theta \right)}\frac{\partial }{\partial \theta_{i}}V\left(\theta \right)=\frac{1}{V\left(\theta \right)}\frac{\partial }{\partial \theta_{i}}{\left\langle \int_{0}^{\infty }e^{-\frac{\tau }{\tau_{e}}}r\left(\tau \right)d\tau \right\rangle }_{p\left(r,z|\theta \right)}$$

can be evaluated using the well-known identity $\frac{\partial }{\partial x}{〈f(a)〉}_{p(a|x)}={〈f(a)\frac{\partial }{\partial x}\log p(a|x)〉}_{p(a|x)}$:

(20) $$\begin{array}{c} \frac{\partial }{\partial \theta_{i}}\log V\left(\theta \right)=\frac{1}{V\left(\theta \right)}{\left\langle \int_{0}^{\infty }e^{-\frac{\tau }{\tau_{e}}}r\left(\tau \right)\frac{\partial }{\partial \theta_{i}}\log p\left(r\left(\tau \right),z\left(\tau \right)|\theta \right)d\tau \right\rangle }_{p\left(r,z|\theta \right)}\\ =\frac{1}{V\left(\theta \right)}{\left\langle \int_{0}^{\infty }e^{-\frac{\tau }{\tau_{e}}}r\left(\tau \right)\frac{\partial }{\partial \theta_{i}}\left(\log p\left(r\left(\tau \right)|z\left(\tau \right)\right)+\log p_{N}\left(z\left(\tau \right)|\theta \right)\right)d\tau \right\rangle }_{p\left(r,z|\theta \right)}\\ ={\left\langle \int_{0}^{\infty }e^{-\frac{\tau }{\tau_{e}}}\frac{r\left(\tau \right)}{V\left(\theta \right)}\frac{\partial }{\partial \theta_{i}}\log p_{N}\left(z\left(\tau \right)|\theta \right)d\tau \right\rangle }_{p\left(r,z|\theta \right)} \end{array}$$

Here, $p_{N}\left(z(\tau )|\text{θ}\right)$ is the probability of observing the spike train z(τ) in the time interval 0 to *τ*. For the definition of the network N given above, the gradient $\frac{\partial }{\partial \theta_{i}}\log p_{N}\left(z(\tau )|\text{θ}\right)$ of this distribution can be directly evaluated. Using Equations 12, 1, we get (Pfister et al., 2006)

(21) $$\frac{\partial }{\partial \theta_{i}}\log p_{N}\left(z\left(\tau \right)|\theta \right)=\frac{\partial w_{i}}{\partial \theta_{i}}\frac{\partial }{\partial w_{i}}\int_{0}^{\tau }z_{{\text{POST}}_{i}}\left(s\right)\log\left(f_{{\text{POST}}_{i}}\left(s\right)\right)-f_{{\text{POST}}_{i}}\left(s\right)ds\approx \int_{0}^{\tau }w_{i}y_{{\text{PRE}}_{i}}\left(s\right)\left(z_{{\text{POST}}_{i}}\left(s\right)-f_{{\text{POST}}_{i}}\left(s\right)\right)ds,$$

where we have used that by construction only the rate function $f_{post_{i}}(s)$ depends on the parameter *θ_i_*. If one discretizes time and assumes that rewards and parameter updates are only realized at the end of each episode, the REINFORCE rule is recovered (Williams, 1992).

In Equation 21, we used the approximation $\frac{\partial w_{i}}{\partial \theta_{i}}\approx w_{i}$. This expression ignores the discontinuity of Equation 1 at *θ_i_* = 0, where the function is not differentiable. In practice we found that this approximation is quite accurate if *θ*_0_ is large enough such that $\exp(\theta_{i}-\theta_{0})$ is close to zero (which is the case for *θ*_0_ = 3 in our simulation). In control experiments, we also used a smooth function $w_{i}=\exp(\theta_{i}-\theta_{0})$ (without the jump at *θ_i_* = 0), for which Equation 21 is exact, and found that this yields results that are not significantly different from the ones that use the mapping Equation 1.

### Online learning

Equation 20 defines a batch learning rule with an average taken over learning episodes where in each episode network responses and rewards are drawn according to the distribution p(r,z|θ). In a biological setting, there are typically no clear episodes but rather a continuous stream of network inputs and rewards and parameter updates are performed continuously (i.e., learning is online). The analysis of online policy gradient learning is far more complicated than the batch scenario, and typically only approximate results can be obtained that however perform well in practice (for discussions, see Seung, 2003; Xie and Seung, 2004).

To arrive at an online learning rule for this scenario, we consider an estimator of Equation 20 that approximates its value at each time *t* > *τ_g_* based on the recent network activity and rewards during time $\left[t-\tau_{g},t\right]$ for some suitable *τ_g_* > 0. We denote the estimator at time *t* by *G_i_*(*t*) where we want $G_{i}(t)\approx \frac{\partial }{\partial \theta_{i}}\log V(\text{θ})$ for all *t* > *τ_g_*. To arrive at such an estimator, we approximate the average over episodes in Equation 20 by an average over time where each time point is treated as the start of an episode. The average is taken over a long sequence of network activity that starts at time *t* and ends at time *t* + *τ_g_*. Here, one systematic difference to the batch setup is that one cannot guarantee a time-invariant distribution over initial network conditions as we did there since those will depend on the current network parameter setting. However, under the assumption that the influence of initial conditions (such as initial membrane potentials and refractory states) decays quickly compared to the time scale of the environmental dynamics, it is reasonable to assume that the induced error is negligible. We thus rewrite Equation 20 in the form (we use the abbreviation $PSP_{i}\left(s\right)=w_{i}\left(s\right)y_{{\text{PRE}}_{i}}\left(s\right)$).

$$\frac{\partial }{\partial \theta_{i}}\log V\left(\theta \right)\approx G_{i}\left(t\right)=\frac{1}{\tau_{g}}\int_{t}^{t+\tau_{g}}\int_{\zeta }^{t+\tau g}e^{-\frac{\tau -\zeta }{\tau_{e}}}\frac{r\left(\tau \right)}{V\left(\theta \right)}\int_{\zeta }^{\tau }PSP_{i}\left(s\right)\left(z_{{\text{POST}}_{i}}\left(s\right)-f_{{\text{POST}}_{i}}\left(s\right)\right)ds\;d\tau \;d\zeta ,$$

where *τ_g_* is the length of the sequence of network activity over which the empirical expectation is taken. Finally, we can combine the second and third integral into a single one, rearrange terms and substitute *s* and *τ* so that integrals run into the past rather than the future, to obtain

(22) $$G_{i}\left(t\right)\approx \frac{1}{\tau_{g}}\int_{t-\tau_{g}}^{t}\frac{r\left(\tau \right)}{V\left(\theta \right)}\int_{0}^{\tau }e^{-\frac{s}{\tau_{e}}}PSP_{i}\left(\tau -s\right)\left(z_{{\text{POST}}_{i}}\left(\tau -s\right)-f_{{\text{POST}}_{i}}\left(\tau -s\right)\right)ds\;d\tau ,$$

We now discuss the relationship between *G_i_*(*t*) and Equations 15, 16 to show that the latter equations approximate *G_i_*(*t*). Solving Equation 15 with zero initial condition *e_i_*(0) = 0 yields

(23) $$e_{i}\left(t\right)=\int_{0}^{t}e^{-\frac{s}{\tau_{e}}}PSP_{i}\left(t-s\right)\left(z_{{\text{POST}}_{i}}\left(t-s\right)-f_{{\text{POST}}_{i}}\left(t-s\right)\right)ds.$$

This corresponds to the inner integral in Equation 22, and we can write

(24) Gi(t) ≈ 1τg∫t−τgt r(τ)V(θ) ei(τ) dτ =〈r(t)V(θ) ei(t)〉τg ≈〈r(t)r^(t) ei(t)〉τg,

where ${〈\cdot 〉}_{\tau_{g}}$ denotes the temporal average from *t*– *τ_g_* to *t* and $\hat{r}(t)$ estimates the expected discounted reward through a slow temporal average.

Finally, we observe that any constant *α* can be added to r(τ)/V(θ) in Equation 20 since

(25) $${\left\langle \int_{0}^{\infty }e^{-\frac{\tau }{\tau_{e}}}\alpha \frac{\partial }{\partial \theta_{i}}\log p_{N}\left(z\left(\tau \right)|\theta \right)d\tau \right\rangle }_{p\left(r,z|\theta \right)}=0$$

for any constant *α* (cf. Williams, 1992; Urbanczik and Senn, 2009).

Hence, we have $G_{i}(t)\;\approx \;{〈\left(\frac{r(t)}{\hat{r}(t)}+\alpha \right)\;e_{i}(t)〉}_{\tau_{g}}$. Equation 16 implements this in the form of a running average and hence $g_{i}(t)\approx G_{i}(t)\approx \frac{\partial }{\partial \theta_{i}}\log V(\text{θ})$ for *t* > *τ_g_*. Note that this result assumes that the parameters θ change slowly on the time scale of *τ_g_* and *τ_g_* has to be chosen significantly longer than the time constant of the eligibility trace *τ_e_* such that the estimator works reliably, so we require $\tau_{e}<\tau_{g}<\frac{1}{\beta }$. The time constant *τ_a_* to estimate the average reward V(θ) through $\tau_{a}\frac{d\hat{r}(t)}{dt}=-\hat{r}(t)+r(t)$ should be on the same order as the time constant *τ_g_* for estimating the gradient. We selected both to be 50 s in our simulations. Simulations using the batch model outlined above and the online learning model showed qualitatively the same behavior for the parameters used in our experiments (data not shown).

### Simulation details

Simulations were preformed with NEST (Gewaltig and Diesmann, 2007) using an in-house implementation of the synaptic sampling model (Kappel D, Hoff M, Subramoney A, 2017); additional tests were run in Matlab R2011b (Mathworks). The code/software described in the paper is freely available online at URL: https://github.com/IGITUGraz/spore-nest-module. The differential equations of the neuron and synapse models were approximated using the Euler method, with fixed time steps Δ*t* = 1 ms. All network variables were updated based on this time grid, except for the synaptic parameters *θ_i_*(*t*) according to Equation 18, which were updated only every 100 ms to reduce the computation time. Control experiments with Δ*t* = 0.1 ms, and 1-ms update steps for all synaptic parameters showed no significant differences. If not stated otherwise synaptic parameters were initially drawn from a Gaussian distribution with *μ* = –0.5 and *σ* = 0.5 and the temperature was set to *T* = 0.1. The learning rate for the synaptic dynamics was chosen to be *β* = 10^–5^ and synaptic delays were 1 ms. Synaptic parameter changes were clipped at ±4 × 10^–4^ and synaptic parameters *θ_i_* were not allowed to exceed the interval [–2, 5] for the sake of numerical stability.

### Details to: Task-dependent routing of synaptic connections through the interaction of stochastic spine dynamics with rewards

The number of potential excitatory synaptic connections between each pair of input and MSNs neurons was initially drawn from a Binomial distribution (*p* = 0.5, *n* = 10). The connections then followed the reward-based synaptic sampling dynamics Equation 5 as described above. Lateral inhibitory connections were fixed and thus not subject to learning. These connections between MSNs neurons were drawn from a Bernoulli distribution with *p* = 0.5 and synaptic weights were drawn from a Gaussian distribution with *μ* = –1 and *σ* = 0.2, truncated at zero. Two subsets of ten neurons were connected to either one of the targets *T*_1_ or *T*_2_.

To generate the input patterns we adapted the method from Kappel et al. (2015). The inputs were representations of a simple symbolic environment, realized by Poisson spike trains that encoded sensory experiences *P*_1_ or *P*_2_. The 200 input neurons were assigned to Gaussian tuning curves (*σ* = 0.2) with centers independently and equally scattered over the unit cube. The sensory experiences *P*_1_ and *P*_2_ were represented by two different, randomly selected points in this 3D space. The stimulus positions were overlaid with small-amplitude jitter (*σ* = 0.05). For each sensory experience the firing rate of an individual input neuron was given by the support of the sensory experience under the input neuron’s tuning curve (maximum firing rate was 60 Hz). An additional offset of 2-Hz background noise was added. The lengths of the spike patterns were uniformly drawn from the interval [750, 1500 ms]. The spike patterns were alternated with time windows (durations uniformly drawn from the interval [1000, 2000 ms]), during which only background noise of 2 Hz was presented.

The network was rewarded if the assembly associated to the current sensory experience fired stronger than the other assembly. More precisely, we used a sliding window of 500 ms length to estimate the current output rate of the neural assemblies. Let ${\hat{\nu }}_{1}(t)$ and ${\hat{\nu }}_{2}(t)$ denote the estimated output rates of neural pools projecting to *T*_1_ and *T*_2_, respectively, at time *t* and let *I*(*t*) be a function that indicates the identity of the input pattern at time *t*, i.e., *I*(*t*) = 1 if pattern *P*_1_ was present and *I*(*t*) = –1 if pattern *P*_2_ was present. If $I(t)({\hat{\nu }}_{1}(t)-{\hat{\nu }}_{2}(t))<0$ the reward was set to *r*(*t*) = 0. Otherwise the reward signal was given by $r(t)=S\left(\frac{1}{5}(I(t){\hat{\nu }}_{1}(t)-I(t){\hat{\nu }}_{2}(t)-\nu_{0})\right)$, where *ν*_0_ = 25 Hz is a soft firing threshold and *S*(⋅) denotes the logistic sigmoid function. The reward was recomputed every 10 ms. During the presentation of the background patterns no reward was delivered.

In Figure 2*D*,*E*, we tested our reward-gated synaptic plasticity mechanism with the reward-modulated STDP pairing protocol reported in Yagishita et al. (2014). We applied the STDP protocol to 50 synapses and reported mean and SEM values of synaptic weight changes in Figure 2*D*,*E*. Briefly, we presented 15 pre/post pairings; one per 10 s. In each pre/post pairing, 10 presynaptic spikes were presented at a rate of 10 Hz. Each presynaptic spike was followed (Δ*t* = 10 ms) by a brief postsynaptic burst of 3 spikes (100 Hz). The total duration of one pairing was thus 1 s indicated by the gray shaded rectangle in Figure 2*E*. During the pairings the membrane potential was set to *u*(*t*) = –2.4 and Equations 14, 15, 16, 18 solved for each synapse. Reward was delivered here in the form of a rectangular-shaped wave of constant amplitude 1 and duration 300 ms to mimic puff application of dopamine. Rewards were delivered for each pre/post pairing and reward delays were relative to the onset of the STDP pairings. The time constants *τ_e_* and *τ_g_*, the reward offset *α* and the temperature *T* of the synapse model were chosen to qualitatively match the results of Yagishita et al. (2014), their Figures 1, 4 (Table 1). The value of *τ_a_* for the estimation of the average reward has been chosen to equal *τ_g_* based on theoretical considerations (see above, Online learning). We found that the parameters of the prior had relatively small effect on the synaptic dynamics on timescales of 1 h.

Synaptic parameter changes in Figure 2*G*were measured by taking snapshots of the synaptic parameter vectors every 4 min. Parameter changes were measured in terms of the Euclidean norm of the difference between two successively recorded vectors. The values were then normalized by the maximum value of the whole experiment and averages over five trials were reported.

To generate the dPCA projection of the synaptic parameters in Figure 2*J*, we adopted the methods of Kobak et al. (2016). We randomly selected a subset of 500 synaptic parameters to compute the projection. We sorted the parameter vectors by the average reward achieved over a time window of 10 min and binned them into 10 equally spaced bins. The dPCA algorithm was then applied on this dataset to yield the projection matrix and estimated fractions of explained reward variance. The projection matrix was then applied to the whole trajectory of network parameters and the first two components were plotted. The trajectory was projected onto the estimated expected reward surface based on the binned parameter vectors.

### Details to: A model for task-dependent self-configuration of a recurrent network of excitatory and inhibitory spiking neurons

Neuron and synapse parameters were as reported above, except for the inhibitory neurons for which we used faster dynamics with a refractory time *t*_ref_ = 2 ms and time constants *τ_m_* = 10 ms and *τ_r_* = 1 ms for the PSP kernel. The network connectivity between excitatory and inhibitory neurons was as suggested previously (Avermann et al., 2012). Excitatory (pools D, U, and hidden) and inhibitory neurons were randomly connected with connection probabilities given in Avermann et al. (2012), their Table 2. Connections include lateral inhibition between excitatory and inhibitory neurons. The connectivity to and from inhibitory neurons was kept fixed throughout the simulation (not subject to synaptic plasticity or rewiring). The connection probability from excitatory to inhibitory neurons was given by 0.575. The synaptic weights were drawn from a Gaussian distribution (truncated at zero) with *μ* = 0.5 and *σ* = 0.1. Inhibitory neurons were connected to their targets with probability 0.6 (to excitatory neurons) and 0.55 (to inhibitory neurons) and the synaptic weights were drawn from a truncated normal distribution with *μ* = –1 and *σ* = 0.2. The number of potential excitatory synaptic connections between each pair of excitatory neurons was drawn from a binomial distribution (*p* = 0.5, *n* = 10). These connections were subject to the reward-based synaptic sampling and rewiring described above. In the resulting network scaffold around 49% of connections consisted of multiple synapses.

To infer the lever position from the network activity, we weighted spikes from the neuron pool D with –1 and spikes from U with + 1, summed them and then filtered them with a long PSP kernel with *τ_r_* = 50 ms (rise) and *τ_m_* = 500 ms (decay). The cue input pattern was realized by the same method that was used to generate the patterns *P*_1_ and *P*_2_ outlined above. If a trial was completed successfully the reward signal *r*(*t*) was set to 1 for 400 ms and was 0 otherwise. After each trial a short holding phase was inserted during which the input neurons were set to 2-Hz background noise. The lengths of these holding phases were uniformly drawn from the interval [1, 2 s]. At the time points marked by an asterisk, the reward policy was changed by switching the decoding functions of the neural pools D and U and by randomly regenerating the input cue pattern.

To identify the movement onset times in Figure 3*D*, we adapted the method from Peters et al. (2014). Lever movements were recorded at a sampling rate of 5 ms. Lever velocities were estimated by taking the difference between subsequent time steps and filtering them with a moving average filter of five-time steps length. A Hilbert transform was applied to compute the envelope of the lever velocities. The movement onset time for each trial was then defined as the time point where the estimated lever velocity exceeded a threshold of 1.5 in the upward movement direction. If this value was never reached throughout the whole trial the time point of maximum velocity was used (most cases at learning onset).

The trial-averaged activity traces in Figure 3*D* were generated by filtering the spiking activity of the network with a Gaussian kernel with *σ* = 75 ms. The activity traces were aligned with the movement onset times (Fig. 3*D*, black arrows) and averaged across 100 trials. The resulting activity traces were then normalized by the neuron’s mean activity over all trials and values below the mean were clipped. The resulting activity traces were normalized to the unit interval.

Turnover statistics of synaptic connections in Figure 3*E* were measured as follows. The synaptic parameters were recorded in intervals of 2 h. The number of synapses that appeared (crossed the threshold of *θ_i_*(*t*) = 0 from below) or disappeared (crossed *θ_i_*(*t*) = 0 from above) between two measurements were counted and the total number was reported as turnover rate.

For the consolidation mechanism in Figure 3*F*, we used a modified version of the algorithm where we introduced for each synaptic parameter *θ_i_* an independent mean *μ_i_* for the prior distribution $p_{S}\left(\text{θ}\right)$. After four simulated days, we set *μ_i_* to the current value of *θ_i_* for each synaptic parameter and the standard deviation *σ* was set to 0.05. Simulation of the synaptic parameter dynamics was then continued for 10 subsequent days.

For the approximation of simulating retracted potential synaptic connections in Figure 3*C*,*G*, we paused evaluation of the SDE (Eq. 5) for *θ_i_* ≤ 0. Instead, synaptic parameters of retracted connections where randomly set to values above zero after random waiting times drawn from an exponential distribution with a mean of 12 h. When a connection became functional at time *t* we set *θ_i_*(*t*) = 10^–5^ and reset the eligibility trace *e_i_*(*t*) and gradient estimator *g_i_*(*t*) to zero and then continued the synaptic dynamics according to Equation 5. Histograms in Figure 3*F* were computed over bins of 2-h width.

**Figure 6.**
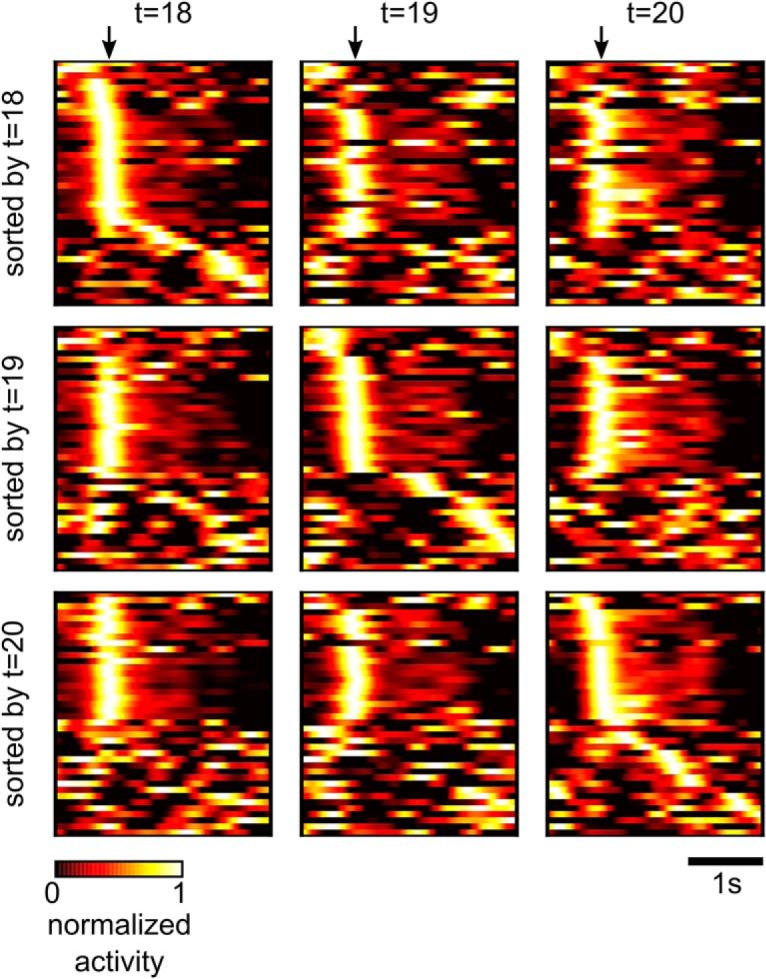
Drifts of neural codes while performance remained constant. Trial-averaged network activity as in Figure 3*D* evaluated at three different times selected from a time window where the network performance was stable (Fig. 3*C*). Each column shows the same trial-averaged activity plot but subject to different sorting. Rows correspond to one sorting criterion based on one evaluation time.

In Figure 6, we further analyzed the trial-averaged activity at three different time points (18, 19, and 20 h) where the performance was stable (Fig. 3*C*). Drifts of neural codes on fast time scales could also be observed during this phase of the experiment.

**Figure 7.**
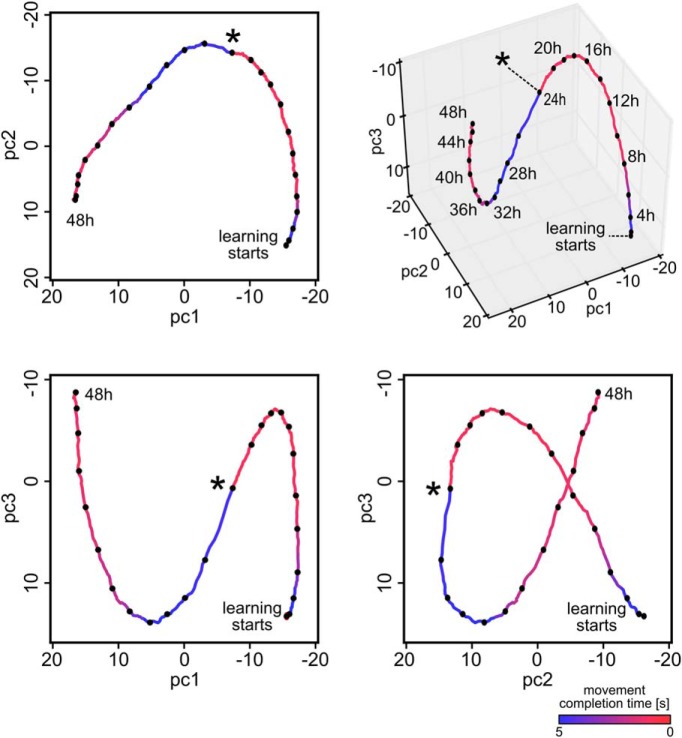
2D projections of the PCA analysis in Figure 3*I*. The 3D projection as in Figure 3*I*, top right, and the corresponding 2D projections are shown.

Since the 3D illustration of the PCA projection in Figure 3*I* is ambiguous, the corresponding 2D projections are shown in Figure 7. The projection to the first two components (pc1 and pc2) show the migration of synaptic parameters to a new region after the task change. The first three principal components explain 82% of the total variance in the parameter dynamics.

### Details to: Compensation for network perturbations

The black curve in Figure 3*C* shows the learning curve of a network for which rewiring was disabled after the task change at 24 h. Here, synaptic parameters were not allowed to cross the threshold at *θ_i_* = 0 and thus could not change sign after 24 h. Apart from this modification the synaptic dynamics evolved according to Equation 18 as above with *T* = 0.1.

For the analysis of synaptic turnover in Figure 3*G*, we recorded the synaptic parameters at *t*_1_ = 24 h and *t*_2_ = 48 h. We then classified each potential synaptic connection *i* into one of four classes, stable nonfunctional: $\left(\theta_{i}(t_{1})\leq 0)\wedge (\theta_{i}(t_{2})\leq 0\right)$, transient decaying: $\left(\theta_{i}(t_{1})>0)\wedge (\theta_{i}(t_{2})\leq 0\right)$, transient emerging: $\left(\theta_{i}(t_{1})\leq 0)\wedge (\theta_{i}(t_{2})>0\right)$, and stable functional: $\left(\theta_{i}(t_{1})>0)\wedge (\theta_{i}(t_{2})>0\right)$.

In Figure 3*H*, we randomly selected 5% of the synaptic parameters *θ_i_* and recorded their traces over a learning experiment of 48 h (one sample per minute). The PCA was then computed over these traces, treating the parameter vectors at each time point as one data sample. The high-dimensional trace was then projected to the first three principal components in Figure 3*H* and colored according to the average movement completion time that was acquired by the network at the corresponding time points.

### Details to: Relative contribution of spontaneous and activity-dependent processes to synaptic plasticity

Synaptic weights in Figure 5*A*,*B* were recorded in intervals of 10 min. We selected all pairs of synapses with common pre- and postsynaptic neurons as CI synapses and synapse pairs with the same post- but not the same presynaptic neuron as non-CI synapses. In Figure 5*D–F*, we took a snapshot of the synaptic weights after 48 h of learning and computed the Pearson correlation of all CI and non-CI pairs for random subsets of around 5000 pairs. Data for 100 randomly chosen CI synapse pairs are plotted of Figure 5*E*.

In Figure 5*F*, we analyzed the contribution of activity-dependent and spontaneous processes in our model. Dvorkin and Ziv (2016) reported that a certain degree of the stochasticity in their results could be attributed to their experimental setup. The maximum detectable correlation coefficient was limited to 0.76–0.78, due to the variability of light fluorescence intensities which were used to estimate the sizes of postsynaptic densities. Since in our computer simulations we could directly read out values of the synaptic parameters we were not required to correct our results for noise sources in the experimental procedure (see pp. 16ff and equations on p. 18 of Dvorkin and Ziv, 2016). This is also reflected in our data by the fact that we got a correlation coefficient that was close to 1.0 in the case *T* = 0 (Fig. 5*D*). Following the procedure of Dvorkin and Ziv (2016), we estimated in our model the contributions of activity history dependent and spontaneous synapse-autonomous processes as in Dvorkin and Ziv (2016), their Figure 8*E*. Using the assumption of zero measurement error and thus a theoretically achievable maximum correlation coefficient of *r* = 1.0. The Pearson correlation of CI synapses was given by 0.46 ±0.034 and that of non-CI synapses by 0.08 ±0.015. Therefore, we estimated the fraction of contributions of specific activity histories to synaptic changes (for *T* = 0.15) as 0.46 – 0.08 = 0.38 and of spontaneous synapse-autonomous processes as 1.0 – 0.46 = 0.54 (Dvorkin and Ziv, 2016). The remaining 8% (measured correlation between non-CI synapses) resulted from processes that were not specific to presynaptic input, but specific to the activity of the postsynaptic neuron (neuron-wide processes).

**Table 1.** Parameters of the synapse model Equations 15, 16, and 18

Symbol	Value	Description
*T*	0.1	Temperature
*τ_*e*_*	1 s	Time constant of eligibility trace
*τ_*g*_*	50 s	Time constant of gradient estimator
*τ_*a*_*	50 s	Time constant to estimate the average reward
*α*	0.02	Offset to reward signals
*β*	10^–5^	Learning rate
*μ*	0	Mean of prior
*σ*	2	STD of prior

Parameter values were found by fitting the experimental data of Yagishita et al. (2014). If not stated otherwise, these values were used in all experiments.

10.1523/ENEURO.0301-17.2018.ed1 Extended Data Supplementary Source Code. Download Extended Data, ZIP file.

## Synthesis

Reviewing Editor: Tatyana Sharpee, The Salk Institute for Biological Studies Decisions are customarily a result of the Reviewing Editor and the peer reviewers coming together and discussing their recommendations until a consensus is reached. When revisions are invited, a fact-based synthesis statement explaining their decision and outlining what is needed to prepare a revision will be listed below. The following reviewer(s) agreed to reveal their identity: NONE. Note: If this manuscript was transferred from JNeurosci and a decision was made to accept the manuscript without peer review, a brief statement to this effect will instead be what is listed below.

The manuscript addresses an important question of how stable network function can be maintained despite continuous changes in synaptic connectivity. The manuscript can be improved by including a more complete discussion of how the new work builds on prior work and how specific features in the model impact its explanatory power. It is especially important to address the novelty of results compared to the prior work by Rokni and colleagues. Furthermore, the link to the Bayesian inference should either be better explained or omitted. Both reviewers provided strong arguments against Bayesian interpretation.

Reviewer 1:

Kappel et al address the question how stable network function can be achieved while synaptic connectivity is undergoing a continuous turnover and even the synaptic weights seem to be primarily determined by synapse-autonomous noise. To answer this question they investigate a synapse dynamics which is composed from a synapse autonomous Ornstein-Uhlenbeck process and a classic reinforcement learning component. They show mathematically that this process will converge to a stationary distribution, which can be interpreted as the posterior distribution of a Bayesian inference framework trying to estimate rewarded network configurations. The synapse autonomous noise term then leads to a sampling of different rewarded network configurations rather than to a convergence to a single optimal configuration. The authors show that, despite the resulting continuous changes of the network configuration, networks converge to and keep a high task performance. This is demonstrated both for Boltzmann machines and recurrent spiking networks, which demonstrates the general applicability of the approach.

Finally and possibly most interestingly, they investigate how the system reacts to a reversal of the trained task. When varying the strength of the noise in their synapse dynamics, they find that the best performance in the relearning task is achieved, when this noise accounts for more than half of the synapse dynamics. Strikingly, these levels are similar to those found in experimental data. Yet, in this context the influence of other model parameters remains elusive.

Although very mathematical, the paper is generally understandable and very well written.

However, a few concerns, which I list in the following, remain to be clarified:

Major points

(1) As I understand, one major point that distinguishes the presented framework from previous reinforcement learning is the existence of the prior. However, the influence of the prior is very poorly investigated/demonstrated. While in Fig. 4 the Temperature, which scales the noise is investigated, the influence of the weighting between prior and reward term or the influence of the parameters of the prior (mean and std) are unclear. Hence it would be good to demonstrate

(a) Different priors lead to different, but equally good solutions (posteriors).

(Especially a demonstration that non-factorizing priors do work would be nice. For example, a ‘homeostatic’ prior which reinforces a constant sum of weights would be an idea for this. For sake of simplicity ths can be done in the Bolzmann machine framework

(b) Does the scaling of reward-term relative to the prior term change the results (e.g. by multiplying the reward and hence its gradient by a constant factor)? Is there a point at which learning fails and the prior dominates the dynamics?

(c) Partly related to (b), how does a change of the prior parameters influence the learning. This would be especially interesting for the rewiring results.

(d) How does the existence of the prior influence the forgetting? As far as I see it, once the reward is taken away, the performance should decay. This issue should at least be discussed in depth as it would limit the biologiacal applicability. Also here the prior parameters will play an important role. Fo a flat prior one would expect a diffusive behaviour while a sharp prior might lead to a fast decay in performance.

(2) The rewiring results at different temperatures (Fig 4d) are very interesting. However, in its current form it is unclear whether the learning has reached a stationary state or not. I would expect that the drop in performance in the low temperature cases is a consequence of unfinished learning. For clarity, I would ask to add traces as in Fig 3c,e for all temperatures. If the traces indeed show, that the optimal temperature is time-dependent, you might want to turn your argument around and use the Dvorkin and Ziv data to determine for which relearning-timescale the synapse dynamics is optimized.

Following points 1b and c, traces for different prior parameters or reward amplitudes would be needed as well. Hence, the optimal working point should be searched in a multidimensional parameter space (compare line 70).

(3) It needs to be better worked out, why a network would want to do Bayesian (policy) sampling of its configurations rather than just adding noise to its dynamics. The mere fact of mathematical equivalence does not give an explanation what this sampling is good for. Is the information about the distribution readout or integrated at some point?

I think that the main arguments are in the text, but must be brought together in a precise manner. Possibly, one needs to introduce the concept of expected expected reward, where the latter expectation refers to the stochastic dynamics of neurons and rewards and the first one refers to the stochasticity of network dynamics.

Also, in a Bayesian framework, there is no free parameter T. Is this a generalisation and should it then still be called Bayesian ?

(4) The motivation of your work by biological plausiblility and realism. I do see that the g-traces, which approximate the reward gradient do only depend on local quantities. Yet, I am not shure about the actual biological implplementation. Could please you discuss shortly, how you think these traces are biologically implemented (e.g. how does a synapse access the firing probabilities or do the low pass filtering). Here, also the relation to the conventional interpretation of the dopamine signal as reward-prediction error (work by Wolfram Schultz) should be discussed a bit more in detail.

Also, please make clear, which timescales and parameters are derived from matching the Yagashita data and which ones are still freely selected. Would learning be possible without the low pass filtering? And: How do the timescale for reward averaging (l.756) and the learning rate for the parameters \beta relate ? I guess at some point the low-pass filtered r starts top pick up rewards from previous parameter configurations.

(5) Fig.2: The task is very well defined here. Can you derive the task relevant and irrelevant dimensions and plot a reward map / sampling trajectory similar as in Fig.1 here?

(6) In the recurrent information routing task: Would it make a difference if the hidden neurons were not experiencing a reward based gradient. As far as I see, the readout neurons should still be able to rout the information based on the reward, even when the representations in the hidden neurons were randomly drifting. Hence, the rewards might reinforce and amplify random changes. Is there a way to evaluate this? Possibly by the correlations of \Delta \theta or by investigating the dynamics when switching off the reward for these neurons?

(7) To see the networks capacity without rewiring, the fixed-connectivity experiment in Fig 3c,e should be repeated with a connectivity, which is fixed from the beginning. Does this also affect the performance of the initial learning?

(8) L.473ff Related to major point 3: I do not see the functional relevance of the Bayesian inference and sampling for the brains computation here. Although the mathematical framework is equivalent to Bayesian inference, there is no readout of the distribution (e.g. a integration over the sampled network configurations) in subsequent layers because, as you show, the relevant network function stays the same. Hence, the sampling is rather on the algorithmic level.

In my opinion, the only functional advantage that you are showing is that the network stays very plastic despite a stable function which allows it to rapidly adapt to task changes (lines 514ff), which has been proposed similarly in Fauth et al 2015. Along this line, you should shortly discuss the relation to the exploitation-exploration dilemma and the plasticity-stability dilemma.

Minor points

L.039 I think the link between growth/shrinkage and rewiring needs to be made clearer.

L.062 You should mention that reinforcement is instrumental to the answer given in this paper.

L.064 As this idea has been described before, you may want to cite e.g. Loewenstein et al 2015 here.

L.064 I would skip on the reference to Fig 1f,2i and 3g, as they have not been explained yet.

L.068 You may want to use “Synapse-autonomous stochastic dynamics”

L.070 ‘immediately’ seems a bit bold when looking at Fig 3c. Also ‘automatically’ is kind of strange. Should';t a learning algorithm adapt automatically anyways? I think the main point here is that usual reinforcement-learning algorithms have problems with unlearning or relearning. If this is the case this should be clarified and proven by a few references.

L.094 I would put the reference to the figure in brackets for a better reading flow.

Fig.1 caption: Please expand on the caption to panel B. At least the title of the axis should be reflected

L.103 I would break the paragraph here.

Eq.2 It is not directly clear that this Equation reflects the Ornstein-Uhlenbeck process mentioned in the preceeding paragraph. It should be noted at some point, that the drift term is absorbed in the deterministic part together with the reward-related terms.

L.133 The jump from an ensemble interpretation (multiple solutions) to the time interpretation (fraction of time) assumes ergodicity an should be explained in more detail.

L.137-143 The explanation of the network scaffold breaks the storyline of the weight dynamics .It might be a good idea to move it to Line 103 or 96.

L.144ff As far as I see it, a plasticity rule is constructed, but it is not shown that these are the only rules. Hence the question is a bit more general than the answers given in the paper. Possibly this sentence can be removed anyway after moving the previous paragraph.

L.146 Possibly this sentence would be better understandable if it was turned around. E.g. “To ensure that the dynamics converge to a stationary distribution p^*, we can choose the deterministic term as ... such that the dynamics becomes ...”

L.173 Check sentence structure.

L.182 You might want to mention that only the rewarded network function is stable.

L.185 I would use all synapses and neurons in the network scaffold

L.188 The integral should be specified better. Is it a temporal or probabilistic integral?

L.195 The connection between the sentences is confusing. Does this still refer to policy gradient learning. If yes, please clarify.

L.199-208 Does this paragraph also still refer to policy gradient learning? The first sentence seems to indicate that this paragraph is unrelated to the previous one. Possibly, it could be left out and the second sentence could be started by, ‘For spiking neurons, ...’.

L.207 The term episode is unclear here. Also, does this sentence refer to the elegibility traces before or to the gradient in general?

L.214 So far, you only explained the sampling part, but not the Bayesian part.

Fig2i: The connections are not really well distinguishable. Maybe a (change in) connection matrix would be a better choice to show this.

L.223 You may want to mention the reward along with the pre and postsynaptic activity.

L.294 How did Peters et al indicate this timewindow?

Fig.3h The shown trajectory cannot really be interpreted. Would it be possible to map it to 2d or show projections on the axial planes? Also, to interpret the meaning of a trajectory in principal component space, it would be nice to know, how much variance the given axis account for.

Fig 3g. I would propose to put the most interesting values for transient emerging and decaying connections at the left and right end, such that different populations can be more directly compared.

L.301 Please explain shortly how the neuronal activities are converted to a lever position. This also helps to interpret the rasterplot etc. In this context, you may want to move the description of the reward dynamics after the description of the network (e.g. l.323)

L.348f During relearning after the task switch, there is quite a significant difference between blue and green curve. I would also expect this from the different distribution of waiting times. Please comment.

L.365 The drifts in neural codes are quite a jump in topic as they are neither mentioned before not in the Xu et al. paper. You may want to use something like “Another experimentally observed phenomenon, that occurs in our model”

l.385 If traces for different temperatures are added, this can be shown elegantly by comparing with the T->0 trace.

L.406 What is the conclusion? Are the synapse autonomous processes comparably stong for this dataset?

L.409 The number of multisynaptic connections is strongly dependent on your potential synapse distribution. Hence, the 49% multi-synaptic should be moved to the methods, when the P-distribution has been described.

L.430 Please specify for which test/hypothesis this p-value was calculated)

L.463f I would leave out the ‘computational’ here

L.485 This was one of your assumptions rather than a prediction.

L.491 Also here: is it a prediction if there is alreadz data on it?

L.516 changes

L.595 I think, here a short discussion of the assumptions in Theorem 1 is necessary. I doubt that the neural dynamics (and hence te reward) always depend on the weights in a continous fashion. For example, the activity in recurrently connected populations undergoes bifurcations when changing the weights. This may lead to a non-differentiable jump in

the population activity once the weight has been increased beyond the critical weight, where the low activity fixed-point vanishes.

L.628 I think this sentence is ab it unclear. Please expand on this.

L.661 Isn';t the eligibility trace multiplied (or, if you will, gated) by the reward?

L.665 You might want to mention here that the next section investigates this alpha in more detail.

L.667 and others: Is it really t>tau_g or rather the timescale of the task, which should not be smaller? Also: Did learning work at all when only using 1s elegibility traces?

L.698 ‘term’

L.761 & L.771 Are these simulations shown anywhere?

L.904 Please expand a bit on how the fractions of contribution are calculated from the correlations.

Reviewer 2:

This manuscript addresses an interesting and important question: synapses are dynamic and change constantly in the living brain. Moreover, while standard learning models posit that synaptic changes are activity-dependent, it seems that much of these dynamics is due to intrinsic, activity-independent processes. This raises the question of how stable functionality is maintained in spite of these synaptic dynamics.

This paper addresses this question by proposing that synaptic dynamics is driven by the sum of three terms (Eq. 5). The third term in the equation is white noise; the second term in the equation is activity- and reward-dependent. It is an implementation of Williams'; REINFORCE learning, which performs gradient ascent on the average reward. The First term is activity-independent. In the absence of reward-dependent learning, the interplay between the first and third terms will determine the stationary distribution of synaptic efficacies. Implementing this rule in a network of spiking neurons, it is demonstrated that functionality is learned and maintained in two tasks, which have been studied experimentally (Figs. 2 and 3). Finally, it is shown that synaptic dynamics is consistent with the experimentally-observed.

While the topic is interesting and the results intriguing, I have some concerns. Please allow me to elaborate:

1) There are important similarities between Rokni, Richardson, Bizzi and Seung, Neuron, 2007 and the current manuscript. The Rokni et al. paper addresses a very similar question of functional stability in the cortex with dynamic, noisy synapses and followed a similar approach. Specifically, Eq. (10) in Rokni et al., which describes synaptic dynamics is composed of the same three terms as Eq. (5) of the current manuscript: a gradient descent learning signal, a white noise term and a decay term. The latter term corresponds to a Gaussian distribution ‘prior’ around zero efficacies, which is the first term in Eq. (5) that determines the stationary distribution in the absence of a reward signal. Nevertheless, Rokni et al. is not mentioned in this manuscript. It is essential that the authors relate to Rokni et al. paper in their manuscript and describe the novelty of this manuscript in view of Rokni et al. paper.

2) The second point, somewhat related to the first point is a more thorough discussion of the novelty in view of other studies on reward-modulated synaptic plasticity. Many previous studies have suggested that synaptic plasticity implements something like stochastic gradient ascent. Any stochastic gradient ascent model, in which the dimensionality of the task is smaller than the dimensionality of the dynamic variables is expected to lead to macroscopic stability in the presence of microscopic dynamics. The reason is that in the synaptic space, there are dimensions, in which the reward is independent of synaptic efficacies. In that sense, functionality in the presence of synaptic dynamics is something that is natural in all these models. The novelty in the current manuscript is the addition of the prior term that adds a (soft) constraint, that shapes the stationary distribution of efficacies, which is undefined in many of those stochastic gradient ascent models. Here it seems that the simple Gaussian prior term (line 220) is equivalent to a decay term. It would have been interesting to go beyond this decay term and study how other functions affect the dynamics of learning and the steady-state distribution of synapses.

3) Specifically, the similarity between the simulations and the experiments is interesting and novel, however, what is lacking is a better explanation of what are the specific features in the model that are critical to explaining what aspect of the experimental results.

4) My next concern is the Bayesian interpretation of Eq. (5). Indeed, the first and second terms in Eq. (5) can be written as a product of two functions, as done in Eq. (4). However, I did not understand the Bayesian interpretation of this product. It is true that for one particular value of T (T=1), the dynamics will converge to the ‘prior’ distribution in the absence of reward. However, beyond this point, I did not understand in what sense the dynamics are performing any sort of Bayesian inference. If not, then the use of the Bayesian formulation is misleading. If yes, then the authors should elaborate on this point

Minor points:

Line 171-175. The naïve reader may be misled to believe that it is generally accepted that OU process underlies synaptic dynamics. In fact, the different papers cited there (and Kaufman et al., 2014 should also be included) all describe different (and inconsistent) dynamics of synapses. Don';t get me wrong. The choice of an OU is reasonable when modeling synapses, in particular given the experimental inconsistency. However, it would be better to mention that OU process is not the generally accepted description of synaptic dynamics.

I also recommend citing Nagaoka et al., Scientific Report, 2016. To the best of my knowledge, this paper is the only direct in vivo study demonstration that synapse dynamics is really autonomous.

Finally, the authors may be interested in two very recent reviews (and references therein) on ‘spontaneous’ synaptic dynamics, which present somewhat opposing views on the matter: Berry and Nedivi, Neuron, 2017 and Mongillo et al., Current Opinion in Neurobiology, 2017.

## References

[B1] Abraham WC, Robins A (2005) Memory retention–the synaptic stability versus plasticity dilemma. Trends Neurosci 28:73–78. 10.1016/j.tins.2004.12.003 15667929

[B2] Avermann M, Tomm C, Mateo C, Gerstner W, Petersen C (2012) Microcircuits of excitatory and inhibitory neurons in layer 2/3 of mouse barrel cortex. J Neurophysiol 107:3116–3134. 10.1152/jn.00917.2011 22402650

[B3] Bartol TM Jr, Bromer C, Kinney J, Chirillo MA, Bourne JN, Harris KM, Sejnowski TJ (2015) Nanoconnectomic upper bound on the variability of synaptic plasticity. Elife 4:e10778. 26618907 10.7554/eLife.10778 PMC4737657

[B4] Baxter J, Bartlett PL (2000) Direct gradient-based reinforcement learning. Proceedings of the 200 IEEE International Symposium on Circuits and Systems, 3:271–274.

[B5] Bellec G, Kappel D, Maass W, Legenstein R (2017) Deep rewiring: training very sparse deep networks. arXiv arXiv:1711.05136.

[B6] Berry KP, Nedivi E (2017) Spine dynamics: are they all the same? Neuron 96:43–55. 10.1016/j.neuron.2017.08.008 28957675 PMC5661952

[B7] Botvinick M, Toussaint M (2012) Planning as inference. Trends Cogn Sci 16:485–488. 10.1016/j.tics.2012.08.006 22940577

[B8] Chaisanguanthum KS, Shen HH, Sabes PN (2014) Motor variability arises from a slow random walk in neural state. J Neurosci 34:12071–12080. 10.1523/JNEUROSCI.3001-13.2014 25186752 PMC4152607

[B9] Chambers AR, Rumpel S (2017) A stable brain from unstable components: emerging concepts, implications for neural computation. Neuroscience 357:172–184. 10.1016/j.neuroscience.2017.06.005 28602920

[B10] Collins AG, Frank MJ (2016) Surprise! dopamine signals mix action, value and error. Nat Neurosci 19:3. 10.1038/nn.4207 26713740

[B11] Deger M, Seeholzer A, Gerstner W (2016) Multi-contact synapses for stable networks: a spike-timing dependent model of dendritic spine plasticity and turnover. arXiv arXiv:1609.05730.

[B12] Desai NS, Rutherford LC, Turrigiano GG (1999) Plasticity in the intrinsic excitability of cortical pyramidal neurons. Nat Neurosci 2:515–520. 10.1038/9165 10448215

[B13] Ding M, Rangarajan G (2004). First passage time problem: a Fokker–Planck approach In: New directions in statistical physics (Wille L, ed), pp 31–46. Berlin: Springer.

[B14] Driscoll LN, Pettit NL, Minderer M, Chettih SN, Harvey CD (2017) Dynamic reorganization of neuronal activity patterns in parietal cortex. Cell 170:986–999. 28823559 10.1016/j.cell.2017.07.021 PMC5718200

[B15] Dvorkin R, Ziv NE (2016) Relative contributions of specific activity histories and spontaneous processes to size remodeling of glutamatergic synapses. PLoS Biol 14:e1002572. 27776122 10.1371/journal.pbio.1002572 PMC5077109

[B16] Fan Y, Fricker D, Brager DH, Chen X, Lu H-C, Chitwood RA, Johnston D (2005) Activity-dependent decrease of excitability in rat hippocampal neurons through increases in *Ih* . Nat Neurosci 8:1542–1551. 10.1038/nn1568 16234810

[B17] Fares T, Stepanyants A (2009) Cooperative synapse formation in the neocortex. Proc Natl Acad Sci USA 106:16463–16468. 10.1073/pnas.0813265106 19805321 PMC2738618

[B18] Fauth M, Wörgötter F, Tetzlaff C (2015) The formation of multi-synaptic connections by the interaction of synaptic and structural plasticity and their functional consequences. PLoS Comput Biol 11:e1004031. 25590330 10.1371/journal.pcbi.1004031 PMC4295841

[B19] Florian RV (2007) Reinforcement learning through modulation of spike-timing-dependent synaptic plasticity. Neural Comput 19:1468–1502. 10.1162/neco.2007.19.6.1468 17444757

[B20] Frémaux N, Sprekeler H, Gerstner W (2010) Functional requirements for reward-modulated spike-timing-dependent plasticity. J Neurosci 30:13326–13337. 10.1523/JNEUROSCI.6249-09.2010 20926659 PMC6634722

[B21] Frémaux N, Sprekeler H, Gerstner W (2013) Reinforcement learning using a continuous time actor-critic framework with spiking neurons. PLoS Comput Biol 9:e1003024. 10.1371/journal.pcbi.1003024 23592970 PMC3623741

[B22] Fusi S, Drew PJ, Abbott L (2005) Cascade models of synaptically stored memories. Neuron 45:599–611. 10.1016/j.neuron.2005.02.001 15721245

[B23] Gardiner C (2004) Handbook of stochastic methods, Ed 3 Berlin: Springer.

[B24] Gerstner W, Kistler WM, Naud R, Paninski L (2014) Neuronal dynamics: from single neurons to networks and models of cognition. Cambridge: Cambridge University Press.

[B25] Gewaltig MO, Diesmann M (2007) NEST (NEural Simulation Tool). Scholarpedia 2:1430. 10.4249/scholarpedia.1430

[B26] Grashow R, Brookings T, Marder E (2010) Compensation for variable intrinsic neuronal excitability by circuit-synaptic interactions. J Neurosci 20:9145–9156. 10.1523/JNEUROSCI.0980-10.2010 PMC2913134 20610748

[B27] Holtmaat A, Svoboda K (2009) Experience-dependent structural synaptic plasticity in the mammalian brain. Nat Rev Neurosci 10:647–658. 10.1038/nrn2699 19693029

[B28] Holtmaat AJ, Trachtenberg JT, Wilbrecht L, Shepherd GM, Zhang X, Knott GW, Svoboda K (2005) Transient and persistent dendritic spines in the neocortex in vivo. Neuron 45:279–291. 10.1016/j.neuron.2005.01.003 15664179

[B29] Holtmaat A, Wilbrecht L, Knott GW, Welker E, Svoboda K (2006) Experience-dependent and cell-type-specific spine growth in the neocortex. Nature 441:979–983. 10.1038/nature04783 16791195

[B30] Izhikevich EM (2007) Solving the distal reward problem through linkage of stdp and dopamine signaling. Cereb Cortex 17:2443–2452. 10.1093/cercor/bhl152 17220510

[B31] Kappel D, Habenschuss S, Legenstein R, Maass W (2015) Network plasticity as Bayesian inference. PLoS Comput Biol 11:e1004485. 10.1371/journal.pcbi.1004485 26545099 PMC4636322

[B32] Kappel D, Hoff M, Subramoney A (2017) IGITUGraz/spore-nest-module: SPORE version 2.14.0 (version v2.14.0). Zenodo CrossRef

[B33] Kasai H, Fukuda M, Watanabe S, Hayashi-Takagi A, Noguchi J (2010) Structural dynamics of dendritic spines in memory and cognition. Trends Neurosci 33:121–129. 10.1016/j.tins.2010.01.001 20138375

[B34] Kasthuri N, Hayworth KJ, Berger DR, Schalek RL, Conchello JA, Knowles-Barley DL, Vzquez-Reina A, Kaynig V, Jones TR, Roberts M, Morgan JL, Tapia JC, Seung HS, Roncal WG, Vogelstein JT, Burns R, Sussman DL, Priebe CE, Pfister H, Lichtman J (2015) Saturated reconstruction of a volume of neocortex. Cell 3:648–661. 10.1016/j.cell.2015.06.054 26232230

[B35] Kirkpatrick J, Pascanu R, Rabinowitz N, Veness J, Desjardins G, Rusu AA, Milan K, Quan J, Ramalho T, Grabska-Barwinska A, Hassabis D, Clopath C, Kumaran D, Hadsell R, (2017) Overcoming catastrophic forgetting in neural networks. Proc Natl Acad Sci USA 114:3521–3526. 28292907 10.1073/pnas.1611835114 PMC5380101

[B36] Kirkpatrick S, Gelatt CD Jr, Vecchi M (1983) Optimization by simulated annealing. Science 220:671–680. 10.1126/science.220.4598.671 17813860

[B37] Kobak D, Brendel W, Constantinidis C, Feierstein CE, Kepecs A, Mainen ZF, Romo R, Qi X-L, Uchida N, Machens CK (2016) Demixed principal component analysis of neural population data. Elife 5:e10989. 27067378 10.7554/eLife.10989 PMC4887222

[B38] Legenstein R, Pecevski D, Maass W (2008) A learning theory for reward-modulated spike-timing-dependent plasticity with application to biofeedback. PLoS Comput Biol 4:e1000180. 18846203 10.1371/journal.pcbi.1000180 PMC2543108

[B39] Loewenstein Y, Kuras A, Rumpel S (2011) Multiplicative dynamics underlie the emergence of the log-normal distribution of spine sizes in the neocortex in vivo. J Neurosci 31:9481–9488. 10.1523/JNEUROSCI.6130-10.2011 21715613 PMC6623170

[B40] Loewenstein Y, Yanover U, Rumpel S (2015) Predicting the dynamics of network connectivity in the neocortex. J Neurosci 35:12535–12544. 10.1523/JNEUROSCI.2917-14.2015 26354919 PMC6605403

[B41] Marder E (2011) Variability, compensation, and modulation in neurons and circuits. Proc Natl Acad Sci USA 108 [Suppl 3]:15542–15548. 21383190 10.1073/pnas.1010674108 PMC3176600

[B42] Marder E, Goaillard J-M (2006) Variability, compensation and homeostasis in neuron and network function. Nat Rev Neurosci 7:563–574. 10.1038/nrn1949 16791145

[B43] Marr D, Poggio T (1976) From understanding computation to understanding neural circuitry. Technical report. Cambridge, MA: Massachusetts Institute of Technology.

[B44] Matsuzaki M, Ellis-Davies GC, Nemoto T, Miyashita Y, Iino M, Kasai H (2001) Dendritic spine geometry is critical for ampa receptor expression in hippocampal ca1 pyramidal neurons. Nat Neurosci 4:1086–1092. 10.1038/nn736 11687814 PMC4229049

[B45] Minerbi A, Kahana R, Goldfeld L, Kaufman M, Marom S, Ziv NE (2009) Long-term relationships between synaptic tenacity, synaptic remodeling, and network activity. PLoS Biol 7:e1000136. 19554080 10.1371/journal.pbio.1000136 PMC2693930

[B46] Mongillo G, Rumpel S, Loewenstein Y (2017) Intrinsic volatility of synaptic connections - a challenge to the synaptic trace theory of memory. Curr Opin Neurobiol 46:7–13. 10.1016/j.conb.2017.06.006 28710971

[B47] Nagaoka A, Takehara H, Hayashi-Takagi A, Noguchi J, Ishii K, Shirai F, Yagishita S, Akagi T, Ichiki T, Kasai H (2016) Abnormal intrinsic dynamics of dendritic spines in a fragile x syndrome mouse model in vivo. Sci Rep 6 10.1038/srep26651 PMC4879559 27221801

[B48] Peters AJ, Chen SX, Komiyama T (2014) Emergence of reproducible spatiotemporal activity during motor learning. Nature 510:263–267. 10.1038/nature13235 24805237

[B49] Peters J, Schaal S (2006). Policy gradient methods for robotics. 2006 IEEE/RSJ International Conference on Intelligent Robots and Systems, pp 2219–2225. Piscataway: IEEE.

[B50] Pfister JP, Toyoizumi T, Barber D, Gerstner W (2006) Optimal spike-timing-dependent plasticity for precise action potential firing in supervised learning. Neural Comput 18:1318–1348. 10.1162/neco.2006.18.6.1318 16764506

[B51] Prinz AA, Bucher D, Marder E (2004) Similar network activity from disparate circuit parameters. Nat Neurosci 7:1345–1352. 10.1038/nn1352 15558066

[B52] Rawlik K, Toussaint M, Vijayakumar S (2013) On stochastic optimal control and reinforcement learning by approximate inference. Proceedings of the twenty-third international joint conference on Artificial Intelligence, pp 3052–3056. Palo Alto: AAAI Press.

[B53] Remme MW, Wadman WJ (2012) Homeostatic scaling of excitability in recurrent neural networks. PLoS Comput Biol 8:e1002494. 10.1371/journal.pcbi.1002494 22570604 PMC3342932

[B54] Rokni U, Richardson AG, Bizzi E, Seung HS (2007) Motor learning with unstable neural representations. Neuron 54:653–666. 10.1016/j.neuron.2007.04.030 17521576

[B55] Rubinski A, Ziv NE (2015) Remodeling and tenacity of inhibitory synapses: relationships with network activity and neighboring excitatory synapses. PLoS Comput Biol 11:e1004632. 26599330 10.1371/journal.pcbi.1004632 PMC4658206

[B56] Rumpel S, Triesch J (2016) The dynamic connectome. e-Neuroforum 7:48–53. 10.1007/s13295-016-0026-2

[B57] Schultz W (2002) Getting formal with dopamine and reward. Neuron 36:241–263. 12383780 10.1016/s0896-6273(02)00967-4

[B58] Schultz W, Dayan P, Montague PR (1997) A neural substrate of prediction and reward. Science 275:1593–1599. 10.1126/science.275.5306.1593 9054347

[B59] Seung HS (2003) Learning in spiking neural networks by reinforcement of stochastic synaptic transmission. Neuron 40:1063–1073. 10.1016/S0896-6273(03)00761-X 14687542

[B60] Statman A, Kaufman M, Minerbi A, Ziv NE, Brenner N (2014) Synaptic size dynamics as an effectively stochastic process. PLoS Comput Biol 10:e1003846. 10.1371/journal.pcbi.1003846 25275505 PMC4183425

[B61] Stettler DD, Yamahachi H, Li W, Denk W, Gilbert CD (2006) Axons and synaptic boutons are highly dynamic in adult visual cortex. Neuron 49:877–887. 10.1016/j.neuron.2006.02.018 16543135

[B62] Sutton RS, Barto AG (1998) Reinforcement learning: an introduction, Vol 1 Cambridge: MIT Press.

[B63] Tang LS, Goeritz ML, Caplan JS, Taylor AL, Fisek M, Marder E (2010) Precise temperature compensation of phase in a rhythmic motor pattern. PLoS Biol 8:e1000469. 20824168 10.1371/journal.pbio.1000469 PMC2930868

[B64] Urbanczik R, Senn W (2009) Reinforcement learning in populations of spiking neurons. Nat Neurosci 12:250–252. 10.1038/nn.2264 19219040

[B65] van Beers RJ, Brenner E, Smeets JB (2013) Random walk of motor planning in task-irrelevant dimensions. J Neurophysiol 109:969–977. 10.1152/jn.00706.2012 23175799

[B66] van Ooyen A, Butz-Ostendorf M (2017) The rewiring brain. San Diego: Academic Press.

[B67] Vlassis N, Ghavamzadeh M, Mannor S, Poupart P (2012) Bayesian reinforcement learning In: Reinforcement learning, pp 359–386. Berlin: Springer.

[B68] Williams RJ (1992) Simple statistical gradient-following algorithms for connectionist reinforcement learning. Mach Learn 8:229–256. 10.1007/BF00992696

[B69] Xie X, Seung HS (2004) Learning in neural networks by reinforcement of irregular spiking. Phys Rev E Stat Nonlin Soft Matter Phys 69:041909. 10.1103/PhysRevE.69.041909 15169045

[B70] Xu T, Yu X, Perlik AJ, Tobin WF, Zweig JA, Tennant K, Jones T, Zuo Y (2009) Rapid formation and selective stabilization of synapses for enduring motor memories. Nature 462:915–919. 10.1038/nature08389 19946267 PMC2844762

[B71] Yagishita S, Hayashi-Takagi A, Ellis-Davies GC, Urakubo H, Ishii S, Kasai H (2014) A critical time window for dopamine actions on the structural plasticity of dendritic spines. Science 345:1616–1620. 10.1126/science.1255514 25258080 PMC4225776

[B72] Yamahachi H, Marik SA, McManus JNJ, Denk W, Gilbert CD (2009) Rapid axonal sprouting and pruning accompany functional reorganization in primary visual cortex. Neuron 64:719–729. 10.1016/j.neuron.2009.11.026 20005827 PMC2818836

[B73] Yang G, Pan F, Gan W-B (2009) Stably maintained dendritic spines are associated with lifelong memories. Nature 462:920–924. 10.1038/nature08577 19946265 PMC4724802

[B74] Yasumatsu N, Matsuzaki M, Miyazaki T, Noguchi J, Kasai H (2008) Principles of long-term dynamics of dendritic spines. J Neurosci 28:13592–13608. 10.1523/JNEUROSCI.0603-08.2008 19074033 PMC2706274

[B75] Ziv NE, Ahissar E (2009) Neuroscience: new tricks and old spines. Nature 462:859–861. 10.1038/462859a 20016588

[B76] Ziv Y, Burns LD, Cocker ED, Hamel EO, Ghosh KK, Kitch L, Gama AE, Schnitzer MJ (2013) Long-term dynamics of CA1 hippocampal place codes. Nat Neurosci 16:264–266. 10.1038/nn.3329 23396101 PMC3784308

